# Parent-reported children’s self-efficacy in linking family resources to preschoolers’ learning dispositions: a mixed-methods study

**DOI:** 10.1186/s41155-026-00397-y

**Published:** 2026-05-19

**Authors:** Tongyao Wang, Haoyu Qu, Peiyue Wu, Xiaoquan Zhu, Bingzhi Wu

**Affiliations:** 1 School of Education, Central China Normal University, Wuhan, China; 2 School of Computer Science and Information Technology, Adelaide University, Adelaide, Australia; 3 School of Psychology, Central China Normal University, Wuhan, China

**Keywords:** Learning dispositions, Parental involvement, Parent-reported children’s self-efficacy, Socioeconomic status, Network analysis

## Abstract

**Background:**

Preschoolers’ learning dispositions are shaped by both family environments and child-level characteristics, but less is known about how these factors operate together in Chinese family contexts. This study examined how parenting self-efficacy, socioeconomic status, parenting arrangements, parental involvement, and parent-reported children’s self-efficacy were associated with preschoolers’ learning dispositions.

**Methods:**

An explanatory sequential mixed-methods design was used. In the quantitative phase, 481 parents from four kindergartens in northern and southern China completed questionnaires on family factors, parental involvement, parent-reported children’s self-efficacy, and children’s learning dispositions. Descriptive statistics, Pearson correlation analyses, hierarchical regression analyses, and network analysis were conducted. In the qualitative phase, semi-structured interviews with 23 purposively selected parents were analyzed using constructivist grounded theory to further interpret the quantitative findings.

**Results:**

Hierarchical regression showed that parent-reported children’s self-efficacy had the strongest association with learning dispositions in the final model, followed by parenting self-efficacy and parental involvement. The association between socioeconomic status and learning dispositions weakened after parental involvement and parent-reported children’s self-efficacy were included. Network analysis further identified children’s self-efficacy and home participation as important bridge nodes connecting family resources, parental involvement, and learning dispositions. Qualitative findings showed that family atmosphere, home-based participation, teacher feedback, and children’s mastery experiences were linked to children’s confidence, curiosity, initiative, and persistence.

**Conclusions:**

Children’s self-efficacy emerged as a central child-level correlate linking family resources and parental involvement with preschoolers’ learning dispositions. The findings suggest that supportive family interactions may be most relevant to learning dispositions when they are translated into children’s observable confidence, initiative, and persistence in everyday learning activities.

**Supplementary Information:**

The online version contains supplementary material available at 10.1186/s41155-026-00397-y.

“Learning dispositions”—the intrinsic psychological tendencies children exhibit during learning, encompassing curiosity, initiative, persistence, creativity, and reflection (Cai, 2015)—constitute a foundational pillar of early childhood development. The preschool years (ages 3–6) represent a critical period during which these dispositions are rapidly formed and consolidated, laying the groundwork for subsequent academic achievement and lifelong learning (Bierman et al., 2008). Yet little is known about how they are associated with family systems outside the social contexts that dominate current evidence. China offers a theoretically important setting for this question because preschool children’s development unfolds at the intersection of strong educational expectations (Chao, 1994), rapid social transformation (Liang & Liu, 2025), and widespread intergenerational caregiving (Xie & Zhou, 2014). These intersecting cultural and structural dynamics create a unique context in which traditional values and modern socioeconomic forces jointly shape the family environments associated with children’s learning dispositions.

Existing research has examined various factors associated with children’s learning dispositions, including parenting self-efficacy (Huang et al., 2023), SES (Feng & Yao, 2023), parenting arrangements (Wang, 2024), and parental involvement (Hu, 2022). However, these studies often examine each influencing factor in isolation, rarely situating them within a unified theoretical framework to systematically analyze their interdependencies (McWayne & Melzi, 2014).

The Hoover-Dempsey and Sandler (1995) model of the parental involvement process provides an appropriate theoretical framework for systematically understanding this issue. Through multiple revisions, this model has evolved into a comprehensive framework comprising six interconnected levels (see Figure S1 in the Supplementary Material), systematically describing the complete pathway from parental involvement motivation (Level 1), parental involvement behaviors (Level 2), parental involvement mechanisms (Level 3), children’s perceptions of parental involvement (Level 4), children’s learning-related characteristics (Level 5), to children’s learning outcomes (Level 6). Guided by this framework, the present study selected key variables aligned with our research questions, rather than incorporating all levels of the model. Specifically, this study selected from Level 1: parenting self-efficacy and family life context factors—including SES and parenting arrangements; from Level 2: parental involvement; from Level 5: parent-reported children’s self-efficacy; and from Level 6: learning dispositions. This selection was based on three considerations: First, the “values, goals, and expectations” dimension from Level 2 was not included as a separate variable because it is conceptually embedded within parenting self-efficacy and is also expressed through the three behavioral dimensions of involvement. Second, the parental involvement mechanisms in Level 3 (e.g., encouragement, modeling, reinforcement) are often difficult to measure accurately through parental self-report questionnaires in daily parenting contexts (Walker et al., 2005). Third, Level 4 (children’s perceptions of parental involvement) requires children to possess considerable metacognitive and introspective abilities, which poses methodological challenges for preschool-aged children (Dix et al., 2007).

Based on the variable selection described above, we further explore the mechanisms underlying the relationship between family factors and learning dispositions. Social cognitive theory (Bandura, 1997) provides a theoretical lens for this inquiry by conceptualizing self-efficacy as a proximal construct that may help explain associations between environmental inputs and behavioral outcomes. Self-efficacy refers to an individual’s belief in their capability to successfully execute a task. For young children, self-efficacy refers to their belief that they can successfully manage learning-related tasks and challenges (Fantuzzo et al., 2004; Schunk & DiBenedetto, 2021). Through this mechanism, children’s family experiences may be reflected in their efficacy-related beliefs and behaviors. This suggests that parent-reported children’s self-efficacy may constitute a critical pathway connecting family factors to learning dispositions. Moreover, previous studies have found that parental involvement (Fantuzzo et al., 2004), parenting self-efficacy (Huang et al., 2023), SES (Borman & Overman, 2004), and parenting arrangements show indirect associations with learning dispositions through parent-reported children’s self-efficacy. Accordingly, this study proposes *Hypothesis 1*: *Parent-reported children’s self-efficacy is positively correlated with learning dispositions and may serve as a proximal pathway linking broader family factors to learning dispositions.*

Within Level 1 (motivational and contextual foundations), Parenting self-efficacy refers to parents’ confidence in their ability to successfully plan, organize, and execute parenting tasks (Montigny & Lacharité, 2005). Grounded in social cognitive theory, parents with higher self-efficacy are more likely to persist in the face of parenting challenges, adopt adaptive coping strategies, and maintain positive patterns of parent-child interaction (Bandura, 1997). Specifically, parents with higher self-efficacy tend to adopt supportive and responsive parenting styles, thereby creating a stimulating learning environment and a positive emotional atmosphere for their children, these two factors are precisely the core parenting conditions essential for the development of learning dispositions (Jones & Prinz, 2005). SES is a key indicator of its parenting conditions, and is typically assessed across the following three dimensions: family income, parental education level, and parental occupation (Bradley & Corwyn, 2002; Ren, 2010). From the perspective of family investment theory, families with higher SES tend to provide children with richer learning resources, higher quality educational opportunities, and broader cultural experiences (Matthews & Gallo, 2011), which may be associated with children’s learning dispositions through daily parenting practices. From the perspective of material resources, higher SES families tend to possess more educational toys, books, and extracurricular activity opportunities—conditions that are theoretically related to children’s curiosity and exploratory behavior (Bradley & Corwyn, 2002). From the perspective of parental education level, more educated parents tend to employ explanatory and dialogic communication styles, an interaction pattern that is positively associated with children’s language development and cognitive persistence (Hoff, 2013). Furthermore, the social networks and time flexibility afforded by occupation enable parents in higher SES families to participate more actively in children’s educational activities, transforming resource advantages into learning support embedded in daily companionship (Matthews & Gallo, 2011). In addition to SES, parenting arrangements, as important contextual conditions, also are also associated with children’s early learning dispositions. In China, grandparent co-parenting is common and often provides practical and emotional support for families, especially when parents face work-related time constraints (Chen et al., 2011). At the same time, from the perspective of family systems theory, when grandparents participate in child-rearing, the family system typically contains more interaction patterns, which may increase relational complexity and lead to inconsistencies in parenting practices (Rothbaum et al., 2002). Regarding the transmission of parenting behaviors, grandparent co-parenting may be negatively associated with learning dispositions through two pathways (Barnett et al., 2012). First, intergenerational disagreements in parenting beliefs may be associated with lower parents’ parenting self-efficacy; when parents’ educational approaches are frequently undermined by grandparents’ experiential knowledge, their consistency and persistence in daily parenting become eroded (Barnett et al., 2012). Second, grandparents’ parenting styles are often shaped by specific historical and cultural backgrounds, emphasizing obedience to rules (Chen & Liu, 2012) while providing relatively less stimulation of curiosity and exploration (Sadruddin et al., 2019). This parenting orientation may create tension with the developmental needs of learning dispositions during the preschool stage. Based on this, this study proposes *Hypothesis 2*: *Family foundational conditions are significantly associated with children’s learning dispositions*,* with positive associations possible for parenting self-efficacy and* SES *and a negative association possible for grandparent co-parenting relative to exclusive parental care.*

Within Level 2 (parental involvement), parental involvement refers to parents’ engagement in a range of educational activities that support children’s healthy development across home, preschool, and community settings, typically encompassing home–kindergarten communication, participation at kindergarten and participation at home (Fantuzzo et al., 2000). Existing research has demonstrated that parental involvement is positively associated with the development of learning dispositions by providing children with learning opportunities and behavioral models that relate to children’s learning beliefs and task persistence (McWayne et al., 2004). Longitudinal studies further indicate that the positive effects of parental involvement on children’s learning engagement remain cross-time stability during the preschool period (El Nokali et al., 2010). Therefore, this study proposes *Hypothesis 3*: *Parental involvement is positively correlated with children’s learning dispositions.*

Based on this integrative framework, the present study systematically examines family- and individual-level factors associated with Chinese preschoolers’ learning dispositions, with particular attention to the role of parent-reported children’s self-efficacy as a key child-level correlate. Specifically, we first examine, through quantitative analysis, whether parent-reported children’s self-efficacy is associated with learning dispositions and compare the strength of this association with factors including parenting self-efficacy, SES, parenting arrangements, and parental involvement. We then employ network analysis to investigate the organizational patterns of these factors within an associative network and identify nodes that play core bridging roles across different variable communities. Finally, through semi-structured interviews, this study provides an in-depth exploration of how parents describe and understand the patterns and possible explanations of these associations in daily family life. By examining how family foundational conditions, parental involvement, and parent-reported children’s self-efficacy are jointly related to preschool children’s learning dispositions, this study aims to extend current understanding of the family processes that support early learning in the Chinese context.

## Method

### Participants and procedure

An explanatory sequential mixed-methods design was employed, with quantitative questionnaire surveys conducted first, followed by qualitative interviews (Creswell & Plano Clark, 2007). In the first phase of this study, we conducted a quantitative survey designed to explore the associations of parenting self-efficacy, SES, parenting arrangements, parental involvement, and parent-reported children’s self-efficacy with learning dispositions. Using convenience sampling, we recruited parents of young children attending four kindergartens to participate voluntarily in the survey. Two kindergartens were located in Xuchang City, Henan Province (Northern China), and two in Liuzhou City, Guangxi Zhuang Autonomous Region (Southern China). After excluding low-quality responses (completion time < 100 s or patterned responding), 481 valid questionnaires were retained from 507 submissions (94.9%) (Credé, 2010). The children had a mean age of 54.83 months. All questionnaires were completed anonymously online by the children’s primary caregivers within one week through the Questionnaire Star platform. Specific sample characteristics are presented in Table 1.

**Table 1:** Demographic information of the participants (*N* = 481)

Variable	Frequency (*n*)	Percentage (%)
Child’s class level		
Lower	189	39.293
Middle	138	28.690
Upper	154	32.017
Child’s gender		
Male	240	49.896
Female	241	50.104
Parenting arrangements		
Parental Parenting	273	56.757
Co-parenting	208	43.243
Other Parenting	0	0
Family income		
< 2000	8	1.663
2001–3000	34	7.069
3001–4000	91	18.919
4001–5000	103	21.414
5001–10,000	180	37.422
10,001–20,000	47	9.771
> 20,000	18	3.742
Occupation		
Temporary/Unskilled/Agricultural labor/Unemployed	18	3.742
Manual workers/Technicians/Self-employed	95	19.751
General/Service/Administrative staff	126	26.195
Middle-level professionals and managers	159	33.056
Senior professionals and managers	83	17.256
Educational Level		
Middle school or below	31	6.445
High school or vocational school	85	17.671
Associate degree	121	25.156
Bachelor’s degree	223	46.362
Master’s degree or above	21	4.366

Co-parenting = parent-grandparent co-parenting; Income = monthly household income per capita (RMB); Occupational status reflects the highest occupational level between the mother and father, based on the highest occupational score according to the occupational classification system; Educational level reflects the highest educational attainment between the mother and father

In the second phase (qualitative interviews), building upon the quantitative findings, this study employed purposive sampling to recruit interview participants, aiming to maximize sample heterogeneity in terms of parental education level, occupation, age, and family structure, with particular attention to the prevalent grandparent co-parenting arrangements. All interviews were face-to-face semi-structured interviews. We first interviewed 19 parents to obtain broad descriptions of children’s daily learning and family life; subsequently, we additionally invited 4 parents to ensure adequate coverage of perspectives and confirm that no new issues emerged. To further explore and clarify key issues identified in the initial interviews, we conducted follow-up interviews with 6 parents from the original sample. Ultimately, a total of 23 parents of young children were interviewed. The basic characteristics of the interviewees are presented in Supplementary Material Table S1. This study received approval from the management departments of the participating kindergartens and the Ethics Committee of the first author’s institution (Approval Number: CCNU-IRB-202409060b), and all participants provided signed informed consent.

### Measures

#### Family socioeconomic status (SES)

SES was operationalized using three standard components: parental occupation, parental educational attainment, and household income (Ren, 2010). Occupational status was coded according to the classification system developed by Shi and Shen (2007), with scores ranging from 1 to 5, ranging from “temporary workers, unemployed individuals, non-skilled workers, and agricultural laborers” to “senior managers, senior professionals, and professional executives”. Parents’ education was coded using six ordinal categories adapted from Li et al. (2020), with values from 1 to 6 corresponding to: primary school or below, junior high school, high school/vocational school, associate degree, bachelor’s degree, and master’s degree or above. Household income was operationalized as monthly per-capita income and classified into seven ordered brackets, scored 1–7: below 2,000 RMB; 2,001–3,000 RMB; 3,001–4,000 RMB; 4,001–5,000 RMB; 5,001–10,000 RMB; 10,001–20,000 RMB; and above 20,000 RMB.

The computation of the composite SES index followed the methodology of Ren, utilizing factor analysis. The extraction results indicated a single dominant component, as only one factor had an eigenvalue exceeding 1. This factor explained 57.748% of the total variance; therefore, subsequent reporting focuses on the loadings of this first principal factor only. The final derived formula is as follows: Family SES = (0.813 × Parental Education + 0.832 × Parental Occupation + 0.616 × Household Income) / 0.577.

#### Parenting self-efficacy (PS-E)

This construct was assessed using the Parenting Self-Efficacy Scale developed by Liu (2014). The instrument includes 19 items across three dimensions: caregiving efficacy (6 items), emotional communication efficacy (7 items), and parenting confidence (6 items). Each item is rated on a five-point Likert scale ranging from 1 (very non-compliant) to 5 (very compliant), with higher scores indicating greater levels of parenting self-efficacy. Sample items include: “I will align with the instructor’s teaching content to provide necessary assistance for the child,” “I can offer timely embraces to the child and articulate words of encouragement,” and “For me, educating children is a fundamentally effortless endeavor.” Evidence also indicates that the instrument performs well in Chinese samples. For instance, Wang and Cheng (2025) reported Cronbach’s alpha values of 0.890, 0.866, and 0.911 for the three subscales, providing further support for its reliability. In the present study, internal consistency was similarly strong: the overall scale yielded a Cronbach’s alpha of 0.948, and the three subscales showed reliability coefficients of 0.889, 0.896, and 0.917, respectively.

#### Family involvement questionnaire-short form

Parental involvement was assessed with the Family Involvement Questionnaire–Short Form (FIQ-SF), first proposed by Fantuzzo et al. (2000) and later adapted for use in China by Liu and Li (2019). Prior evidence with Chinese parent samples indicates that the Chinese revision shows sound psychometric performance, supporting its cultural appropriateness and research utility in Chinese settings. For example, Liu and Li (2019) conducted confirmatory factor analyses and reported excellent fit for both fathers (χ²/*df* = 1.51, RMSEA = 0.04, GFI = 0.96, CFI = 0.99, TLI = 0.98) and mothers (χ²/*df* = 1.75, RMSEA = 0.04, GFI = 0.95, CFI = 0.98, TLI = 0.97). Reliability results were also satisfactory, with Cronbach’s *α* coefficients ranging from 0.88 to 0.94 and test–retest reliability estimates between 0.38 and 0.59 across dimensions. The instrument contains 20 items covering three domains: home–kindergarten communication, participation at kindergarten, and participation at home. Illustrative items include attending parent–teacher meetings to discuss a child’s performance, helping organize educational activities in the kindergarten, and taking children to learning-oriented sites such as zoos or museums. Items are rated on a 4-point Likert scale (1 = rarely, 4 = always), with higher scores reflecting greater involvement; dimension scores are computed as the mean of items within each domain. In the current study, internal consistency was strong (overall Cronbach’s *α* = 0.948), and the three subscales yielded α values of 0.949, 0.902, and 0.906, respectively.

#### Parent-reported children’s self-efficacy

Parent-reported children’s self-efficacy was assessed using the Children’s Self-Efficacy Scale, originally developed by Schwarzer and Jerusalem (1995) and later revised by Huang and Yang (2015). Previous research has supported its cultural appropriateness for Chinese samples. For example, Lin and Ye (2020) reported satisfactory internal consistency (Cronbach’s *α* = 0.818) and strong composite reliability (CR = 0.819, 95% CI: 0.802–0.836), indicating that the instrument is suitable for use in the Chinese context. The scale is unidimensional and consists of six items rated on a 5-point Likert format ranging from “strongly disagree” to “strongly agree.” The items assess children’s confidence when facing challenging or unfamiliar situations, with sample expressions such as “I can do it” and “No problem” when encountering new tasks. Higher scores reflect stronger parent-reported self-efficacy in children. In the present study, the scale demonstrated high reliability, with Cronbach’s *α* = 0.892.

#### Parent rating scale of preschoolers’ learning dispositions

This study adopted the Parent Rating Scale of Preschoolers’ Learning Dispositions, developed by Cai (2015), as the primary assessment instrument. This scale has been widely used and empirically validated among Chinese preschool children. In a study by Lin and Ye (2020), the scale demonstrated excellent reliability and validity, with a total Cronbach’s alpha coefficient of 0.953, composite reliability of 0.891, and a 95% confidence interval ranging from 0.882 to 0.900. The scale comprises 41 items across five dimensions: Creativity and Innovation, Initiative, Curiosity and Interest, Persistence and Attention, and Reflection and Explanation. Sample items include “enjoys making their own toys,” “dares to attempt difficult and challenging activities,” “enjoys observing fish or ants and can maintain this interest for an extended period,” “can refocus attention on the original activity after being distracted,” and “can recognize their own mistakes and explain the reasons.” It is designed to provide a comprehensive assessment of preschoolers’ learning dispositions. For scoring, responses are rated on a 4-point Likert scale, with “Always” assigned 4 points and “Never” assigned 1 point. All items are scored in a positive direction except Item 31 (“The teacher reports that the child has difficulty concentrating in class”), which is coded reversely. In this study, Cronbach’s alpha values for the five subscales were 0.915, 0.908, 0.865, 0.821, and 0.867, respectively, and the scale demonstrated excellent overall reliability (*α* = 0.960), indicating strong internal consistency.

### Data analysis

SPSS 25.0 and R 4.4.3 were used for all quantitative data analyses. First, Harman’s single-factor test was conducted to examine common method bias (Podsakoff et al., 2003). Second, descriptive statistics and Pearson correlation analyses were performed to present the means, standard deviations, and correlation matrices of all variables. To examine the independent associations of each factor with children’s learning dispositions, this study employed hierarchical regression analysis, incorporating variables in three steps according to the hierarchical logic of the parental involvement process model: Model 1 included parenting self-efficacy, SES, and parenting arrangements; Model 2 added parental involvement; Model 3 added parent-reported children’s self-efficacy. To further explore the complex patterns of associations among parenting self-efficacy, SES, parenting arrangements, parental involvement, parent-reported children’s self-efficacy, and learning dispositions, this study conducted network analysis using R 4.4.3. Gaussian Graphical Models (GGM) were estimated to visualize the structural relationships among variables, bridge centrality indices (e.g., bridge expected influence) were calculated, and stability tests were performed to identify core nodes that play connecting roles across different variable communities. For the dichotomous variable, parenting arrangements were coded as 0 = exclusive parental care, 1 = grandparent co-parenting.

During qualitative data analysis, NVivo 12 Plus software served as the primary data management tool. The entire analytical process strictly adhered to the core principles of constructivist grounded theory (Charmaz, 2014). This methodological stance recognizes that meaning is co-constructed through the interaction between researchers and participants, while permitting existing theoretical perspectives to inform interpretation without imposing constraints. To enhance analytical rigor, multiple quality control measures were implemented across various coding stages. Two researchers first independently reviewed all interview transcripts to develop initial codes, concurrently documenting the evolution of their interpretative insights through memo-writing. Regular analysis meetings were convened, during which the coding team compared emerging conceptual patterns, deliberated on divergent interpretations, and ultimately reached consensus on the coding framework. Based on this framework, initial codes were further synthesized and refined into higher-level categories. Throughout this process, category boundaries were continuously optimized through iterative cross-case comparisons. Theoretical sampling strategies guided the selection of supplementary interview materials, aimed at testing and enriching the developing categories until all properties reached saturation. The finalized coding scheme was applied to the entire dataset. In a double-blind coding test on a randomly selected subsample, the inter-coder agreement coefficient reached 93%. All coding discrepancies were traced back to the original transcripts for individual verification and resolved through collective discussion.

## Results

### Common method variance

Before the main analyses, we assessed whether common method variance might threaten the validity of the findings. Using Harman’s single-factor test, the unrotated factor solution yielded 13 factors with eigenvalues exceeding 1. The first factor accounted for 37.693% of the total variance, which falls below the commonly used 40% criterion (Podsakoff et al., 2003). Therefore, the results suggest that common method bias is unlikely to be a serious concern in the present study.

### Descriptive statistics and correlational analysis

Table 2 summarizes the descriptive statistics for the study variables, reporting their means and standard deviations, as well as the associations among them. Bivariate relationships among parenting self-efficacy, SES, parenting arrangements, parental involvement, and parent-reported children’s self-efficacy were examined using Pearson’s correlation coefficients.

**Table 2:** Descriptive statistics and correlations among main variables

Variables	1	2	3	4	5	6
1.Parenting self-efficacy	-					
2.SES	0.188^***^	-				
3.Parenting arrangements	-0.085	0.054	-			
4.Parental involvement	0.604^***^	0.223^***^	-0.089^*^	-		
5.Parent-reported children’s self-efficacy	0.607^***^	0.165^***^	-0.169^***^	0.492^***^	-	
6. Learning Dispositions	0.716^***^	0.252^***^	-0.102^*^	0.669^***^	0.715^***^	-
*M* ± *SD*	4.032 ± 0.529	0.000 ± 3.000	0.430 ± 0.496	2.652 ± 0.627	3.508 ± 0.747	2.875 ± 0.467
Min-Max	2.000–5.000	-10.411–6.785	0.000–1.000	1.200–4.000	1.500–5.000	1.366–4.000

*N* = 481. ^*^p < 0.05, ^***^p < 0.001; *SES* family socioeconomic status; parenting arrangements were dummy coded (0 = parental parenting, 1 = parent–grandparent co-parenting); *M* ± *SD* = mean ± standard deviation; Min-Max=maximum value - minimum value

The correlation matrix indicates that parenting self-efficacy is positively and significantly related to SES (*r* = 0.188, *p* < 0.001), parental involvement (*r* = 0.604, *p* < 0.001), and parent-reported children’s self-efficacy (*r* = 0.607, *p* < 0.001). SES also shows significant positive associations with parental involvement (*r* = 0.223, *p* < 0.001) and parent-reported children’s self-efficacy (*r* = 0.165, *p* < 0.001). In addition, parental involvement is significantly correlated with parent-reported children’s self-efficacy (*r* = 0.492, *p* < 0.001).

In contrast, parenting arrangements exhibit small but significant negative correlations with parental involvement (*r* = -0.089, *p* < 0.05), parent-reported children’s self-efficacy (*r* = -0.169, *p* < 0.001), and learning dispositions (*r* = -0.102, *p* < 0.05), whereas its relationships with the remaining variables are non-significant. Finally, learning dispositions are strongly and positively associated with parenting self-efficacy (*r* = 0.716, *p* < 0.001), parent-reported children’s self-efficacy (*r* = 0.715, *p* < 0.001), and parental involvement (*r* = 0.669, *p* < 0.001), and they are also modestly correlated with SES (*r* = 0.252, *p* < 0.001).

### Factors associated with preschoolers’ learning dispositions

To further examine the independent associations of each factor with children’s learning dispositions after controlling for other variables, this study employed hierarchical regression analysis. Prior to the regression analysis, multicollinearity among the variables was tested. The variance inflation factors (VIF) for all independent variables were below the conventional threshold of 10, with a maximum value of 1.972 (Field, 2024), indicating no serious multicollinearity issues.

As shown in Table 3, all three regression models achieved statistical significance: Model 1 (*F* = 178.517, *p* < 0.001), Model 2 (*F* = 184.229, *p* < 0.001), and Model 3 (*F* = 216.938, *p* < 0.001), indicating that each set of variables significantly explained variance in children’s learning dispositions. The R² values progressively increased from 0.529 in Model 1 to 0.695 in Model 3, demonstrating the enhanced explanatory power of the models as more variables were included.

**Table 3:** Hierarchical regression analyses of factors associated with learning dispositions (N = 481)

	Variables	Model 1	Model 2	Model 3
Level 1	Parenting self-efficacy	0.688^***^	0.482^***^	0.298^***^
	SES	0.126^***^	0.084^**^	0.068^**^
	Parenting arrangements	-0.050	-0.033	0.011
Level 2	Parental involvement		0.356^***^	0.286^***^
Level 5	Parent-reported children’s self-efficacy			0.385^***^
R^2^		0.529	0.608	0.695
Adjusted R^2^		0.526	0.604	0.692
∆R^2^		0.529	0.079	0.088
F (p)		178.517^***^	184.229^***^	216.938^***^
∆F (p)		178.517^***^	95.388^***^	137.089^***^

**p < 0.01, ***p < 0.001. β = the standardized coefficient. The headings Level 1, 2, and 5 refer to the theoretical levels of predictors as defined by the Hoover-Dempsey model of parental involvement, and are not equivalent to the statistical modeling steps (Model 1, 2, 3). Co-parenting = parent-grandparent co-parenting families, with parenting families serving as the reference group; the dependent variable is learning dispositions

In Model 1, parenting self-efficacy showed the strongest positive association with learning dispositions (*β* = 0.688, *p* < 0.001), and SES was also significantly positively associated (*β* = 0.126, *p* < 0.001), while parenting arrangements were not significantly associated with learning dispositions (*p* > 0.05). After incorporating parental involvement in Model 2, parental involvement was significantly positively associated with learning dispositions (*β* = 0.356, *p* < 0.001), and parenting self-efficacy remained strongly associated (*β* = 0.482, *p* < 0.001). The association coefficient for SES diminished but remained significant (*β* = 0.084, *p* < 0.01), while parenting arrangements remained non-significant (*p* > 0.05). In the final model (Model 3), after adding parent-reported children’s self-efficacy, the model’s explanatory power further improved (*R*² = 0.695). Notably, parent-reported children’s self-efficacy showed the strongest positive association with learning dispositions (*β* = 0.385, *p* < 0.001), while parenting self-efficacy (*β* = 0.298, *p* < 0.001) and parental involvement (*β* = 0.286, *p* < 0.001) remained significantly positively associated. The association coefficient for SES, while still significant, was relatively weaker (*β* = 0.068, *p* < 0.01), and parenting arrangements remained non-significant (*p* > 0.05).

Overall, the hierarchical regression results showed that parenting self-efficacy, parental involvement, and parent-reported children’s self-efficacy were the variables most closely associated with young children’s learning dispositions. In the final model, parent-reported children’s self-efficacy showed the strongest standardized association, even after SES, parenting arrangements, parenting self-efficacy, and parental involvement were controlled. Across the stepwise models, the standardized coefficient for SES decreased, whereas parenting arrangements remained nonsignificant.

### Network analysis results

**Fig. 1:**
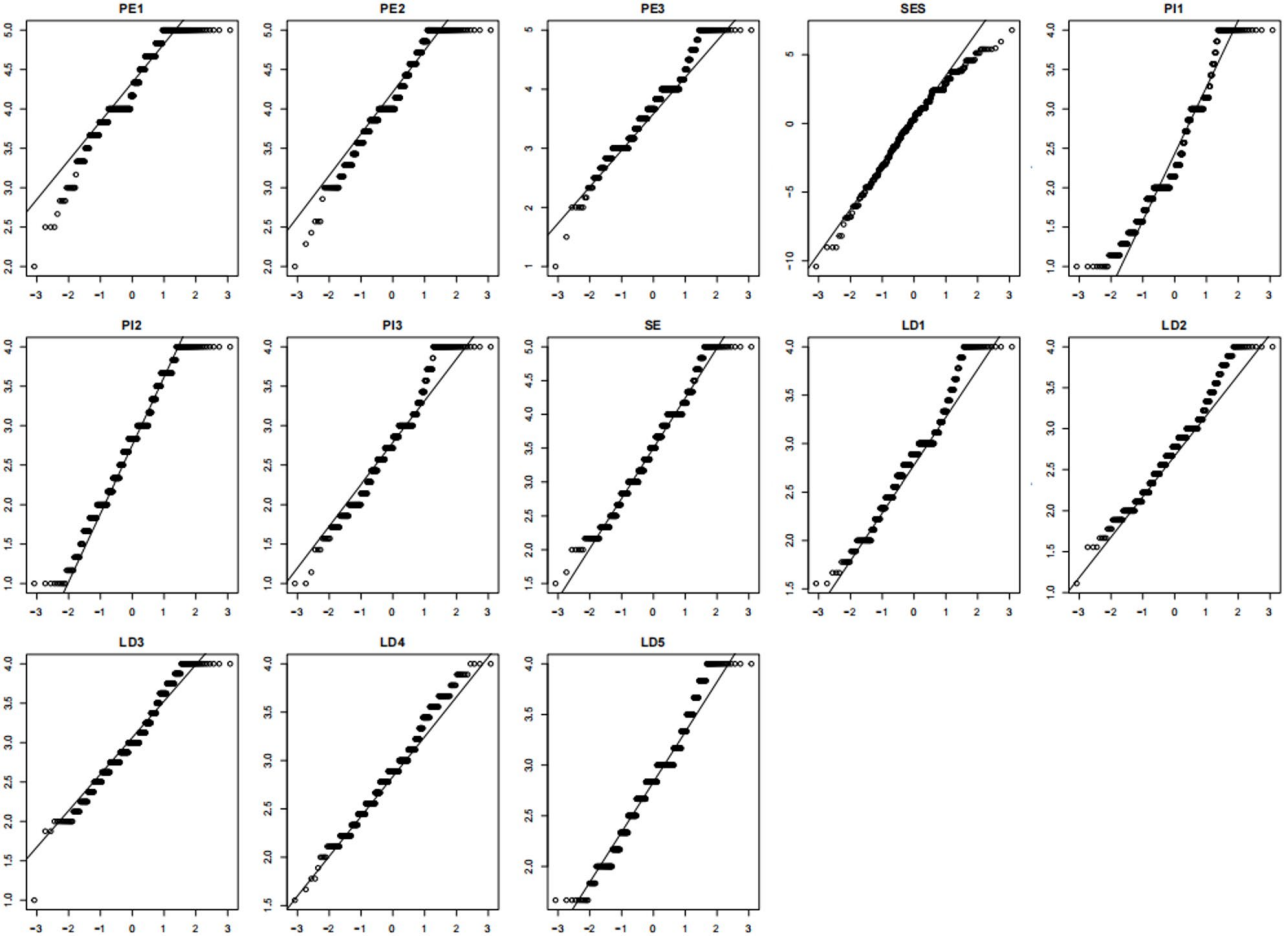
Normal Q–Q plots for the 13 continuous variables used in the GGM

Specifically, a Gaussian graphical model (GGM) was fitted with the EBICglasso procedure implemented in the qgraph package, using a tuning parameter of γ = 0.02. This specification was treated as the main network used for visualization (Fig. 1) and subsequent centrality and bridge-centrality analyses. The resulting adjacency matrix is provided in Supplementary Material Table S2. To address the distributional prerequisite for the continuous variables, we inspected the 13 continuous nodes: univariate skewness and kurtosis were small (|skew| ≤ 0.52; |kurtosis| ≤ 0.64; *N* = 481), and Fig. 1 indicated approximate normality. The binary parenting arrangements variable was treated as ordinal; mixed correlations were computed with cor_auto (polychoric for ordinal pairs). As a robustness check against departures from normality, we re-estimated the network after a rank-based nonparanormal (NPN) transformation; the edge-weight pattern was virtually identical to the main model (edge-weight correlation *r* = 0.991). Taken together, these checks indicate that normality is reasonable for the continuous variables and that our GGM results are robust to minor non-normality.

The network was constructed based on regularized partial correlations among 14 variables, controlling for all other variables. Edges with weights below 0.01 were omitted to enhance interpretability. Each node represented a theoretically distinct construct, categorized into four pre-defined communities: situational and motivating factors (5 nodes), parental involvement (3 nodes), parent-reported children’s self-efficacy (1 node), and learning dispositions (5 nodes). The communities and their corresponding variable abbreviations are presented in Table 4. Nodes were positioned using a spring layout algorithm. In the network, green edges represent positive relationships, whereas red edges denote negative relationships. Edge weights reflect the magnitude of regularized partial correlations, quantifying conditional dependencies.

To evaluate the structural importance of each node, we calculated centrality metrics such as strength and expected influence. Betweenness and closeness were also obtained for reporting purposes; however, given their documented instability in psychological networks (CS = 0), these two indices were not used for substantive interpretation. To further examine nodes’ connecting functions across communities, bridge centrality measures (bridge strength and bridge expected influence) were estimated with the networktools package. In addition, we examined the robustness of the network by testing the stability of edge weights and centrality estimates through nonparametric bootstrapping and case-dropping bootstrapping implemented in the bootnet package, using 1,000 bootstrap replications per node. The correlation stability (CS) coefficient was reported to indicate the dependability of inferences drawn from the centrality results.

### Network estimation

**Fig. 2:**
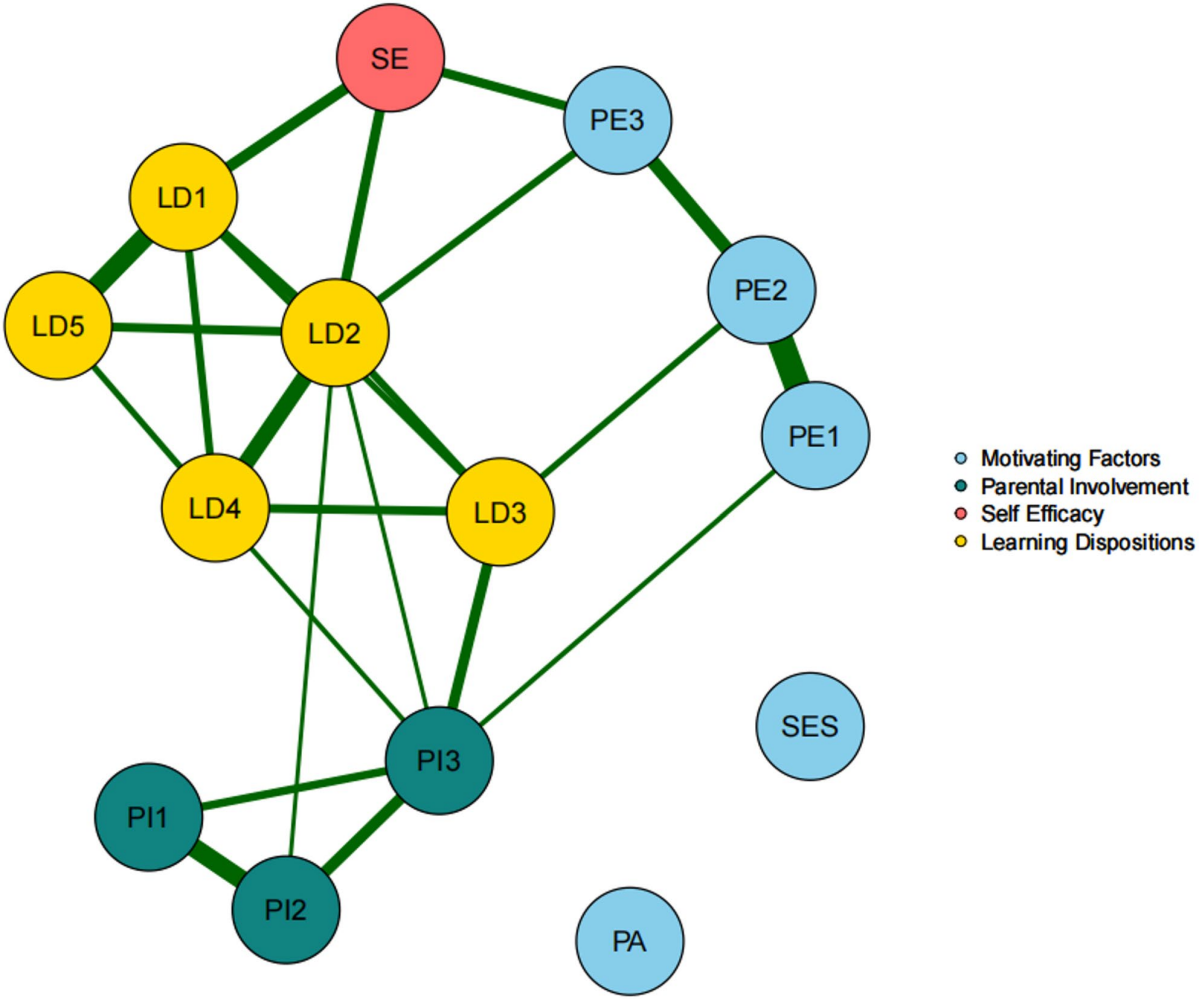
Network diagram of situational and motivating factors, parental involvement, parent-reported children’s self-efficacy, and learning dispositions

Figure 2 presents the EBICglasso depicting conditional associations among situational and motivating factors, parental involvement, parent-reported children’s self-efficacy, and learning dispositions. The final network consisted of 14 nodes. The strongest partial correlation was observed between PE1 and PE2 (edge weight = 0.58), indicating a robust positive association between these two motivational constructs. The Learning Dispositions cluster showed internal coherence, although none surpassed the strength of the PE1-PE2 edge.

Thus, the network did not simply reproduce bivariate correlations; rather, it showed which associations remained after all other variables were conditioned on. The strong PE1–PE2 edge reflects coherence among parenting-efficacy dimensions, whereas the weaker direct edges involving SES and parenting arrangements indicate that these distal family conditions were not central once proximal motivational and involvement variables were considered.

### Node centrality indices

**Fig. 3:**
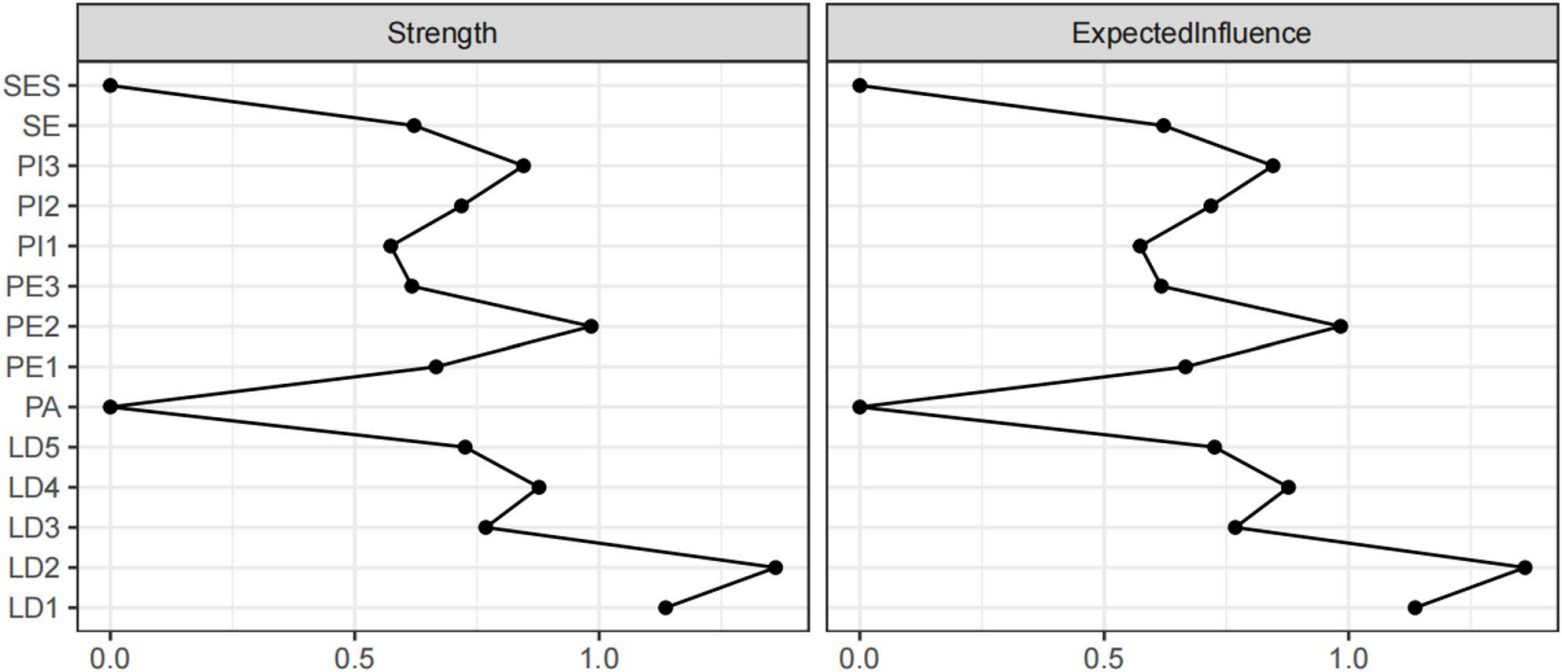
Centrality plot

Figure 3 displays the centrality indices for the 14 nodes in the estimated network. Among all nodes, LD2 exhibited the highest strength (1.36) and expected influence (1.36), indicating that it had more and stronger direct associations with other variables. In contrast, SES and PA showed values of zero for both strength and expected influence, consistent with their absence of connections in the network model and indicating their limited direct roles in the multivariate system after regularization. Both strength and expected influence showed high stability (CS = 0.751 > 0.25), supporting their interpretability (Epskamp et al., 2018).

### Bridge centrality indices

**Fig. 4:**
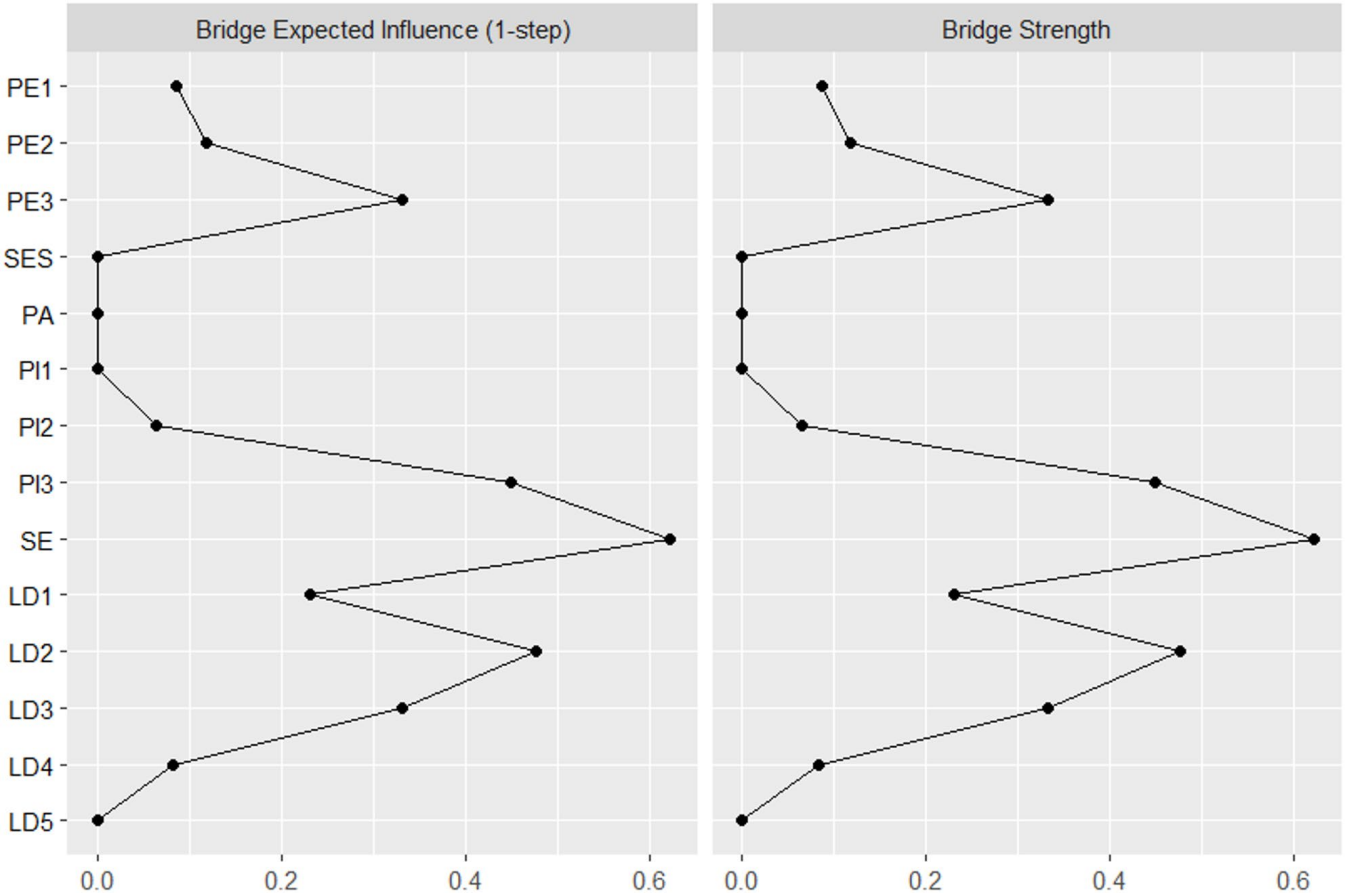
Bridge centrality indices of the network

Figure 4 presents the bridge centrality results. Among all nodes, SE showed the largest bridge strength (0.62) as well as the highest bridge expected influence (0.62), suggesting that it serves as a key connector between otherwise separated components of the network (Jones et al., 2021). In addition, LD2 and PI3 exhibited comparatively strong bridging capacity, with bridge strength values of 0.48 and 0.45, respectively, implying that they help integrate the communities representing situational/motivational factors, parental involvement, parent-reported children’s self-efficacy, and learning dispositions. To examine the robustness of these findings, we assessed the stability of bridge expected influence via a case-dropping bootstrap procedure with 1,000 resamples. The correlation stability coefficient (CS) reached 0.518, which is above the suggested minimum of 0.25, providing support for the credibility of the bridge-centrality interpretations (Epskamp et al., 2018). The corresponding stability pattern across subsamples is illustrated in Figures S2 in the Supplementary Material (Jones et al., 2021).

These bridge-centrality results specify the integrative role of parent-reported children’s self-efficacy. A high bridge expected influence means that this node connected the family/resource and involvement communities to the learning-disposition community through positive conditional associations, making it a plausible associative pattern in which family experiences are connected with observable learning dispositions. The bridging roles of LD2 and PI3 further suggest that children’s initiative and home-based participation are the most direct behavioral carriers of this pathway.

### Network stability

Centrality stability. Case-dropping bootstrap results are presented in Figures S2 and S3 in the Supplementary Material (x-axis = proportion of cases retained; y-axis = average correlation with the full-sample estimates). Bridge expected influence showed acceptable stability (CS ≈ 0.518, Figure S3), above the recommended minimum of 0.25 and near the preferred 0.50 benchmark (Epskamp et al., 2018). In the same figure, node strength and expected influence were highly stable (CS strength ≈ 0.751; CS EI ≈ 0.751). Even when only 50% of the sample was retained, the correlations with the full-sample estimates remained ≈ 0.95 for strength and ≈ 0.94 for expected influence, whereas betweenness had CS = 0, so we do not interpret it (Epskamp et al., 2018). Taken together, these findings suggest that centrality-based interpretations—derived from strength and expected influence—remain stable even when a substantial proportion of cases is removed, which is consistent with prevailing guidelines for psychological network research.

To further examine the robustness of the estimated network (Borsboom et al., 2021), we conducted a nonparametric bootstrap analysis with 1,000 resamples; the results are displayed in Figure S4 in the Supplementary Material (edge-weight accuracy and stability). Figure S4 presents the nonparametric bootstrap assessment of edge weights (1,000 resamples): red dots/lines are the sample estimates, gray ribbons are the 95% CIs, and edges are ordered from strongest (top) to weakest (bottom). Across the network the CIs are generally narrow and largely overlapping, indicating precise edge estimates. Importantly, all edges involving the parenting arrangements indicator (PA) and socioeconomic status (SES) were regularized to zero (Degree = 0), and their CIs straddle zero, whereas cross-community connectivity is instead carried by nodes such as parent-reported children’s self-efficacy (SE) and Learning-Disposition items (e.g., Bridge Strength: SE = 0.621, LD2 = 0.476, PI3 = 0.449, LD3 = 0.332). Robustness checks further confirmed the stability of the edge weights (Epskamp et al., 2018): when the EBIC tuning parameter was varied from the main specification (γ = 0.02) to γ = 0.25 and γ = 0.50, the resulting edge-weight patterns remained virtually unchanged (upper-triangle correlations with the γ = 0.02 solution *r* ≈ 0.997 in both cases). In addition, re-estimating the network after a nonparanormal rank transformation produced a highly similar edge structure (*r* = 0.991). Overall, the findings indicate that the network’s edge estimates are both precise and robust, and they also show that the apparent weak link between learning disposition and parenting arrangements and SES arises because PA and SES do not form reliable edges in the regularized network, while links between learning dispositions and parent-reported children’s self-efficacy/parental involvement items are the ones that persist with tight confidence bands.

Thus, the network did not simply reproduce bivariate correlations; rather, it showed which associations remained after all other variables were conditioned on. The strong PE1–PE2 edge reflects coherence among parenting-efficacy dimensions, whereas the weaker direct edges involving SES and parenting arrangements indicate that these variables had limited direct connectivity within the estimated network.

### Results of constructivist grounded theory

**Fig. 5:**
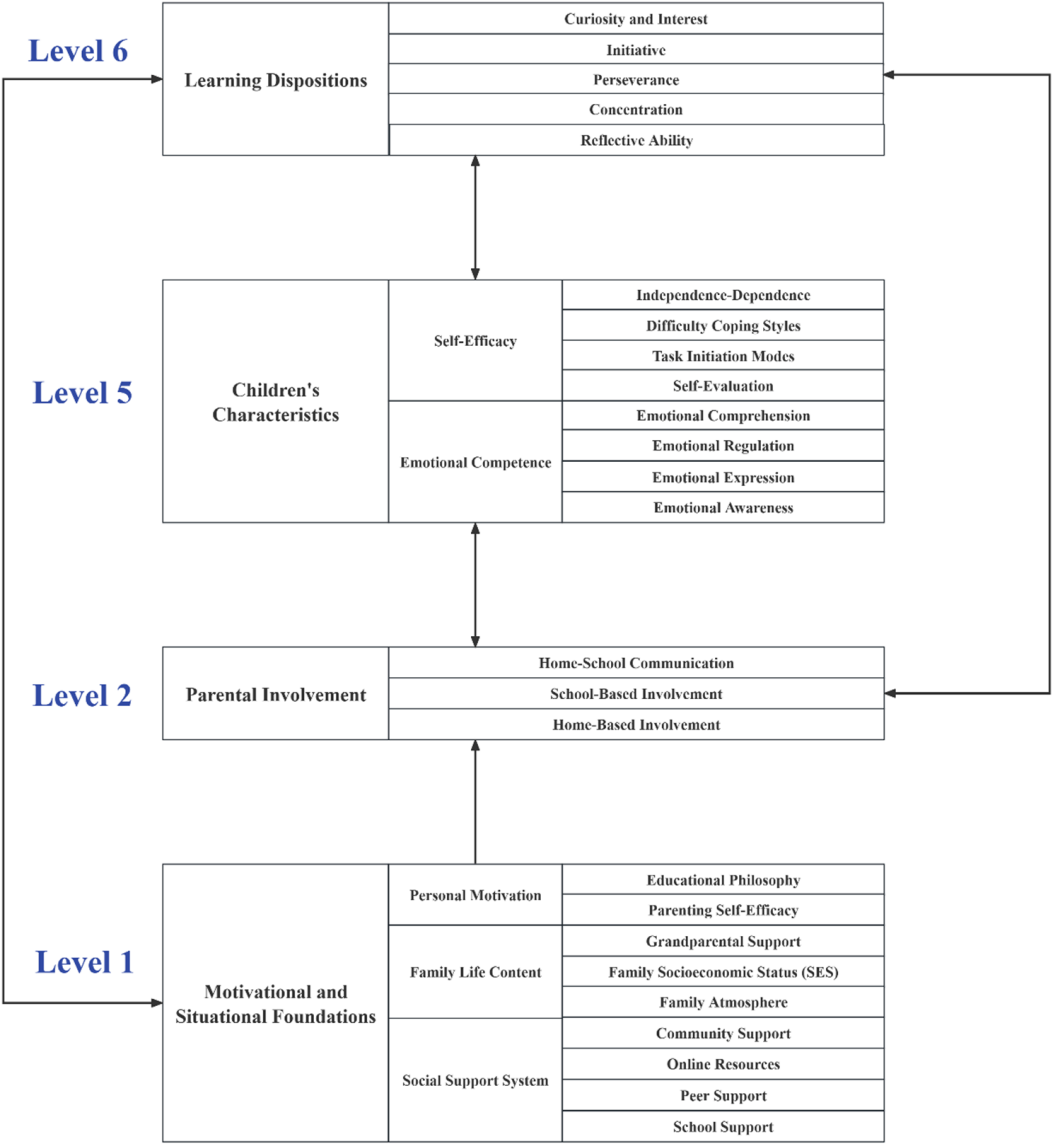
A process model linking parental involvement, parent-reported children’s self-efficacy, and learning dispositions

To deeply interpret and contextualize the quantitative findings, this study employed constructivist grounded theory methods (Charmaz, 2014), utilizing the Hoover-Dempsey model of the parental involvement process as a sensitizing framework to conduct hierarchical coding and constant comparative analysis on the interview data from 23 parents of young children. Building upon the patterns of associations among factors revealed by the quantitative study, this study constructed, through qualitative analysis, a process model of how parental involvement in Chinese families relates to young children’s learning dispositions (see Fig. 5), aiming to explain how these associations concretely occur and unfold in daily life contexts. This analysis generated a total of 62 initial codes, which were integrated into 19 focused codes, and ultimately distilled into four hierarchical levels of core categories. The open coding framework is presented in Supplementary Material Table S3, and the axial coding structure is provided in Supplementary Material Table S4. An integrated joint display synthesizing the quantitative and qualitative findings is available in Supplementary Material Table S5. The model situates parental involvement practices within a progressive system comprising Motivational and Situational Foundations (Level 1), Forms of Parental Involvement (Level 2), Child Characteristics (Level 3), and Learning dispositions (Level 4), and identifies bidirectional regulatory relationships within and between each level. Thus, it provides deep behavioral and meaning-level interpretations for the quantitative findings.

The qualitative findings are presented in a sequence that parallels the quantitative results. Specifically, they first describe how family resources were related to parental involvement, then explain how parental involvement was linked to efficacy-related child characteristics, and finally show how these characteristics were expressed in children’s learning dispositions.

#### Baseline resources: motivational and situational foundations, and parental involvement

Parents usually understand children’s learning quality within a “resource baseline” covering two domains.

#### Motivational and situational foundations

Parents’ understanding of their daily involvement is always rooted in a foundational level composed of personal motivation, family life content and social support system. This level provides value orientation, ability beliefs and realistic constraints for subsequent involvement behaviors, and was described as being associated with children’s learning quality through different paths.

Personal motivation was coded and summarized into two dimensions: educational philosophy and parenting self-efficacy. Educational philosophy mainly includes expectations for children’s cultivation goals and understanding of the initiative in learning. Some parents believe that learning interest and habits are more important than academic performance: *“I don’t advocate sitting at the desk and studying rigidly all the time; I hope he can develop learning interest and form good learning habits at this stage.”* (T3) While other parents emphasize that learning is children’s own business and that children should be allowed to explore independently to promote the development of initiative: *“Except for art classes*,* he wants to take all other courses on his own. He often wants to learn things well because he thinks his mother will be happy if he does*,* and he also feels happy when he learns by himself.”* (T1) Parenting self-efficacy is closely associated with children’s learning dispositions. Parents with high efficacy are full of confidence in their ability to guide children’s learning, and this help children persist and succeed:*“I think I am quite confident in educating children. For example*,* when he encounters difficulties and wants to give up*,* I won’t do it for him directly*,* but will stay with him to figure out solutions together. Some time ago*,* he was learning to skip rope and got anxious because he couldn’t do it well all the time. I accompanied him to practice for ten minutes every day*,* and a week later he really could skip more than a dozen times in a row. He felt a great sense of accomplishment*,* and now he is willing to try more times when facing other things.”* (T1) On the contrary, parents with low parenting efficacy often feel powerless when facing children’s learning difficulties: *“To be honest*,* I have little confidence in teaching children. When he asks me questions*,* I often don’t know how to answer them*,* for fear of misleading him with wrong answers. I find that now when he encounters difficulties*,* the first thing he does is not to figure out solutions by himself*,* but to give up directly or ask others for help*,* which may be related to my lack of guidance skills.”* (T5).

Family life content was coded and summarized into three structural characteristics, which constitute the constraints and underlying conditions for parental involvement: grandparental support, SES, and family atmosphere. The association between grandparental support and children’s learning quality appeared to vary with their role positioning in family education and the power relationship with parents. On the one hand, when grandparents take on auxiliary affairs such as daily care, they free up time and energy for parents to engage in high-quality companionship, thus supporting the development of children’s curiosity, interest and initiative. A parent mentioned: *“Grandpa and grandma are mainly responsible for picking up and dropping off the child and cooking*,* which provides us with more time to accompany the child.”* (T2) On the other hand, when grandparents intervene in the educational process and have conflicting ideas with parents, parents described children’s sense of rules and persistence as being less stable. A parent mentioned that grandpa stepped in to stop the discipline of the child, and the parent described the child as becoming less persistent when encountering difficulties: *“When I discipline the child*,* grandpa will stop me and say*,* ‘He is still young*,* don’t be so strict.’ Now the child has learned to hide behind grandpa and grandma as soon as he makes a mistake*,* and gives up and waits for others’ help when doing something a little difficult.”* (T16).

SES, encompassing parental occupational type, educational level, and household income, influences the development of children’s learning dispositions through its association with parents’ available time and energy, cognitive capacities, and material resources. Parents’ occupational nature determines the time and energy available for companionship. A parent mentioned that their work is regular and they can spend more time accompanying the child: *“Now I spend more time on the child*,* accompanying her to paint and do handcrafts*,* and this kind of companionship is more conducive to her learning quality.”* (T5) In contrast, parents with busy work frankly stated that it is difficult to guarantee the quality of companionship: *“We used to think reading was very important*,* but we are very tired after work*,* so we end up being perfunctory when telling stories.”* (T14) Parents’ educational level affects their ability to understand and respond to children’s developmental needs. A parent with a postgraduate degree mentioned: *“Because I have a high educational level*,* I have a clearer understanding of the child’s developmental stage and learning needs in kindergarten. For example*,* when the teacher said he has recently become interested in numbers*,* I will design more math games in daily life. He is very happy when he figures out the answers*,* and this interest gradually turns into his habit of active exploration.”* (T11) Family income level affects the learning resources and experience opportunities available to children: *“We signed him up for Lego and swimming classes*,* which cost a lot of money a year*,* but we can see he is really interested. He is happy every time he goes*,* and will take the initiative to share what he has learned with us when he comes back. We think it is well worth it.”* (T14).

Family atmosphere was described by parents as the emotional tone, interaction mode and relationship quality permeating daily family life, which constitutes the psychological foundation for the development of children’s learning quality: *“The atmosphere in our family has always been relaxed*,* and we chat about the happy things of each day during dinner every day. Growing up in this environment*,* she has a particularly cheerful personality and is willing to try anything. For example*,* there was a story-telling competition in the kindergarten*,* and she signed up on her own initiative and said*,* ‘I want to have a try.’ Although she didn’t perform the best in the end*,* she was not discouraged at all and said she would participate next time. ”* (T1).

The social support system was coded and summarized into four structural characteristics, which were described as indirectly related to children’s learning quality by providing parents with information, experience and resources: community support, school support, peer support and online resources. Community support (such as community libraries and public welfare courses) not only provides parents with opportunities for external exploration: *“The community women’s federation offers courses*,* and the community and library also organize some activities.”* (T14), but also enables children to contact a broader knowledge field and peer groups, stimulating their interest in new things through diverse experiences: *“There is a Sunny Flower Program and a reading corner in the community*,* which allow her to contact more things.”* (T14) School support is mainly reflected in the kindergarten’s educational philosophy and teachers’ professional attention. A parent mentioned that the reason for choosing this kindergarten is the recognition of its educational philosophy and the experience of teachers’ care for children: *“I specially sent my child to study here. The teachers here pay close attention to the development of each child*,* not only teaching knowledge*,* but also focusing on cultivating habits and interests*,* which is more conducive to the cultivation of children’s learning quality.”* (T12) Peer support is reflected in the communication with other parents. A parent mentioned that when the child showed fear of difficulties in learning, she was originally very anxious, but found it a common phenomenon after communicating with parents of children of the same age: *“My child shouts ‘I can’t do it’ as soon as he encounters a difficult problem. I was very anxious at first*,* and then I talked with other mothers and found that their children are the same. Moreover*,* some mothers shared their experience*,* saying that more encouragement and patient companionship will help children persist on their own gradually. I tried this for a period of time*,* and now although he still hesitates when encountering difficult problems*,* he won’t give up directly and will say ‘I’ll try again’.”* (T12) Notably, online resources have a dual role, suggesting complex associations with learning quality. On the one hand, online resources provide parents with a convenient information access, enabling them to obtain diverse parenting methods and educational resources, thus supporting children’s learning and development in a more targeted way: *“I will browse Douyin and Xiaohongshu to see how other parents teach their children math and character recognition. Some methods are really useful*,* making children interested and willing to learn actively.”* (T16) On the other hand, when parents fail to lead by example, they face an authority crisis in the media management of children, which parents perceived as being related to children’s concentration. A parent reflected: *“We adults like to take mobile phones when going to the toilet*,* but I don’t allow him to play games on mobile phones. Then he will retort*,* ‘Why can mom look at the mobile phone when going to the toilet?’ Now he is also easily distracted when doing things*,* and wants to play other things after reading for a few minutes. I think it may be related to our failure to set a good example.”* (T5).

#### Parental involvement

Parents transform resources and motivation into practices through three specific forms, which act on the formation of children’s learning quality in different ways: participation at home, participation at kindergarten, and home–kindergarten communication.

In participation at home, some parents stimulate children’s active exploration through parent-child reading: *“I read ‘Father and Son’ to him*,* and he will look up what ‘azure blue’ means and ask ‘why’.”* (T14) In participation at kindergarten, parents can grasp children’s learning characteristics more accurately by observing their performance in the collective, and then provide more targeted support at home: *“I went to watch his class on the open day and found that he is actually very willing to raise his hand to answer questions in class*,* which is completely different from at home. This made me realize that he is not uninterested in learning*,* but just needs a suitable atmosphere. Now when I accompany him to study*,* I also imitate the teacher’s way*,* giving more encouragement and interaction*,* and he is indeed more willing to try actively than before.”* (T10) In home–kindergarten communication, some parents adjust their family education strategies according to the specific methods provided by teachers, and children show stronger initiative in learning: *“I asked the teacher what to do if the child can’t remember the characters he has learned*,* and the teacher taught me the method of playing games with character cards. I tried it for a month*,* and now he has learned a lot of characters and will take the initiative to pull me to play the game.”* (T6).

#### Motivational and situational foundations and parental involvement transform into learning quality through parent-reported children’s self-efficacy

Axial coding and relational coding, participants repeatedly presented a core path: most of the time, the motivational and situational foundations at the family level and parental involvement behaviors do not directly act on learning quality, but first become reflected in children’s observable efficacy-related and emotional behaviors, which then relate to learning dispositions. In this study, these two key characteristics were coded as parent-reported children’s self-efficacy and emotional competence, among which parent-reported children’s self-efficacy acts as a key observed child-level indicator linking external support to learning dispositions, and emotional competence serves as an important supporting condition for children’s observable efficacy-related behaviors.

At the motivational and situational foundations level, parents’ parenting efficacy was described as being conveyed through daily interaction, with parents observing that children showed greater efficacy-related confidence and were more willing to try actively when facing learning tasks later:*“I think the most important thing in educating children is to believe that they can learn. When he was learning to ride a balance bike*,* he didn’t want to learn after falling several times*,* and I said to him*,* ‘You will definitely make it after more practice.’ Later he really learned it*,* and said proudly*,* ‘I knew I could do it.’ Now when facing new things*,* he will first say to himself*,* ‘Have a try*,* I should be able to do it.’”* (T17) The reading atmosphere in family life was described by parents as supporting children’s observable confidence in reading, such as saying “I can read by myself,” over time, which is further manifested as the interest and exploratory behavior of active character recognition: *“The child’s father will read books and do exercises in the living room*,* and I read books too*,* so the child will take books by himself and sit with her parents. She has developed a reading habit with us since she was a child*,* and now she will take the initiative to ask when she encounters unfamiliar characters and try to spell them by herself. She will say proudly to me*,* ‘Mom*,* I know this character’ when she spells it correctly.”* (T8).

At parental involvement level, participation at home through daily interactions was described as helping children accumulate successful experiences and display stronger efficacy-related confidence, which in turn drives the development of their learning dispositions:*“I take time to play math games with him in daily life every day*,* such as asking him to count the total number of things bought when going to the supermarket and count the number of fruits for each person when dividing fruits. He is very happy when he calculates the right answer and says*,* ‘Mom*,* I figured it out.’ This happiness is not because of my praise*,* but because he feels he has mastered it*,* the feeling of ‘I can do it’. Now he will take the initiative to calculate when he sees numbers and says*,* ‘I like math because I can calculate the right answers.’”* (T11) Parents described positive feedback from teachers in home–kindergarten communication as strengthening children’s observable efficacy-related confidence in specific fields, making them show stronger initiative and engagement in subsequent activities. A parent said: *“The teacher will take the initiative to tell me about her performance this week*,* such as making progress in painting and doing good handcrafts. I will tell her when she gets home*,* ‘The teacher praised you and said you paint very carefully.’ She is very happy to hear that*,* will be more engaged in painting next time*,* and will take the initiative to say*,* ‘Mom*,* look at my painting*,* is it good?’”* (T1).

Emotional stability enables children to sustain concentration and persistent effort when confronted with learning challenges: *“When in a positive emotional state*,* she would remain calm and deeply engaged for extended periods even when struggling with puzzles*,* demonstrating sustained focus and persistence”* (T8).

#### Practical forms of learning quality

Learning quality in parents’ narratives is a multi-dimensional composite concept highly consistent with the theoretical dimensions of Cai (2015). It not only includes the initial curiosity and interest (*“He is particularly interested in cars*,* dinosaurs and other things*,* and will read books and look at pictures by himself.”* (T6); “When I read ‘Father and Son’ to him, he will look up relevant information and ask ‘why’.” (T14), but also emphasizes the initiative driven by intrinsic motivation (*“He now takes the initiative to say ‘I want to learn’ and will try by himself.”* (T6); *“He now takes the initiative to have oral conversations with me and express himself actively.”* (T2), as well as *persistence* (*“She wants to learn painting*,* so I let her learn*,* and there will be progress if she persists for a period of time.”* (T9) and concentration when facing difficulties (*“He can stick to one thing for a period of time*,* and his attention is better than before.”* (T7); *“If the habits are well persisted during this period*,* his overall state of concentration will be better.”* (T14). Particularly importantly, some parents have also keenly observed children’s reflective ability: *“Sometimes he will say ‘I know why’ and think by himself.”* (T14).

#### Inter-category relationships: relational coding and process direction

The model not only identifies four levels, but also reveals the complex dynamic correlations between them through relational coding. Parents not only described how their behaviors were related to children’s learning states, but also recounted how children’s states were also described as being associated with changes in parents’ concepts, attitudes, and behaviors.

When children demonstrate a more focused and self-disciplined approach to learning, parents often perceive this as validation of their educational efforts, which in turn enhances their parenting confidence and motivates them to seek out additional resources to further support their child’s development. When children show a more serious learning attitude, parents will feel that their educational methods are effective, which not only enhances parents’ parenting confidence: *“Recently*,* he has been much more focused on doing homework and can sit still by himself. Looking at his serious look*,* I think it is still useful for me to accompany him to do exercises and set rules for him at ordinary times*,* and I have to keep doing this in the future.”* (T17), but also makes parents more willing to take the initiative to seek and use online resources: *“Now he knows to finish his homework first and then read books when he comes home every day*,* without my reminder at all. Seeing the child so self-conscious*,* I am also more willing to browse Douyin and Xiaohongshu to see what good learning methods other parents have*,* and want to learn more to accompany him to learn better.”* (T13).

As children exhibit greater initiative and autonomy in their learning, parents become more inclined to invest time and energy in companionship and to maintain active communication with teachers, creating a positive feedback loop that sustains and deepens their involvement. When children show stronger initiative in learning, parents are more willing to invest time and energy in companionship. A parent said: *“Seeing him so self-conscious*,* I am also more motivated to accompany him to do handcrafts and read picture books*,* thinking of spending more time with him while he is willing to learn.”* (T13) When children show a good learning state, parents are more willing to communicate with teachers actively, forming a positive cycle: *“Recently*,* the teacher always praises him for listening carefully in class and answering questions actively. I am very happy to hear that*,* and am also more willing to take the initiative to ask the teacher about his performance in the kindergarten*,* wanting to know what aspects he does well in so that I can continue to encourage him at home.”* (T20).

When children completed challenging tasks with sustained concentration, parents described this mastery experience as being followed by stronger observable confidence, persistence, and willingness to try independently. A parent mentioned: *“When he was building Lego*,* he pondered over a difficult part for half an hour by himself*,* and finally built it successfully. He ran over to me excitedly and said*,* ‘Mom*,* look*,* I figured it out by myself!’ Since then*,* he won’t easily ask for help when encountering a little difficult puzzles*,* and will try by himself first and say*,* ‘I should be able to do it.’”* (T22).

Children’s characteristic states were described as being associated with the nature and intensity of parental involvement: when parents perceive children as showing strong efficacy-related confidence, parents are more likely to adopt a facilitative, hands-off approach, whereas when children exhibit emotional vulnerability in the face of difficulty, parents respond by increasing their provision of patience and guided support. When parents perceive children as displaying confidence that they “can do well,” they are more inclined to let children explore independently and continue to participate as companions at the same time: *“He can arrange his time by himself now*,* and will plan the courses to take*,* homework to finish and books to read by himself when he comes home at night. I just need to sit beside him.”* (T1) When children have weak emotional competence and are prone to give up due to setbacks, parents need to invest more patience in guidance. A parent described: *“He is easy to get anxious when he encounters difficult problems*,* and says he doesn’t want to learn as soon as he gets anxious. At this time*,* I have to stop*,* comfort his mood first*,* and then accompany him to think slowly when he calms down.”* (T19).

#### Divergent and negative cases

To define the boundary conditions of the model and test the robustness of the proposed theoretical relationships, we systematically examined the cases inconsistent with the dominant patterns in the four levels.

The first category: some parents have clear educational goals for their children, but due to busy work, they have limited time for companionship, which affects the development of their children’s curiosity, interest and persistence: *“I think a healthy body and the ability to cooperate and communicate with others are more important than various historical and humanistic knowledge*,* but I work for too long and have no time to accompany him. I don’t know if this is the reason*,* but now he is unwilling to try new things and loses his temper and stops playing when he can’t build blocks well.”* (T3).

The second category: when parents lack educational confidence, even with relatively superior conditional foundations, their children may still have insufficient development in active exploration and independent problem-solving. A parent admitted: *“My work is quite flexible and I have no problem with time*,* but to be honest*,* I have little confidence in teaching children. When he asks me questions*,* I often don’t know how to answer them. Later*,* when he encounters difficult problems*,* the first thing he does is not to figure out solutions by himself*,* but to shout ‘Mom*,* I can’t do it’ directly.”* (T5).

The third category: when parents’ perception and interpretation of children’s states deviate from their actual performance, even if children have shown good concentration and exploratory desire, their learning quality may still be inhibited by parents’ over-intervening participation at home. A parent frankly said: *“He can ponder over building blocks and puzzles for a long time by himself*,* but I just think he can’t sit still. The teacher said he is distracted in class*,* so I am anxious and stare at him to do exercises every day*,* and force him to do it when he doesn’t want to.”* (T19).

The fourth category: a small number of cases show that even if home–kindergarten communication, a form of involvement, is relatively limited, children can still develop the learning quality of active planning and independent arrangement by virtue of early childcare experience: *“I am not good at communicating with teachers unless there is something really important. But he has been going to a childcare center since he was one year and ten months old*,* and now he will plan the courses to take*,* homework to finish and books to read by himself when he comes home at night.”* (T11).

Overall, the qualitative findings showed that parent-reported children’s self-efficacy was described as closely related to mastery experiences, emotional support, and feedback loops among parents, teachers, and children. Negative cases further showed that family resources were not consistently accompanied by stronger children’s efficacy-related behaviors when parental confidence, time, or interpretations of children’s behavior were limited.

## Discussion

Integrating the quantitative and qualitative findings, this study suggests that parent-reported children’s self-efficacy functioned as a key proximal child-level indicator linking parental involvement with young children’s learning dispositions in Chinese families. In the quantitative analyses, parent-reported children’s self-efficacy maintained a strong association with learning dispositions after family socioeconomic resources, parenting arrangements, parenting self-efficacy, and parental involvement were considered, indicating that children’s observable efficacy-related behaviors accounted for variance beyond more distal family conditions. The qualitative findings helped clarify this pattern by showing that parents did not describe these efficacy-related behaviors as isolated child traits; rather, they connected children’s confidence, initiative, curiosity, and persistence to repeated mastery experiences, emotional support, encouragement, and feedback from parents and teachers. In this sense, parental involvement may be linked to learning dispositions partly through the child-level behaviors that parents perceive as self-efficacy, consistent with Hypothesis 1 and with social cognitive theory, which emphasizes the role of environmental support in shaping efficacy-related action patterns (Bandura, 1997). At the same time, the interview data also qualified this pathway: in negative cases, family resources did not consistently translate into stronger learning dispositions when parents lacked confidence, time, or shared interpretations of children’s learning needs. Therefore, parent-reported children’s self-efficacy should be understood not as a standalone internal trait directly measured from children, but as an observed efficacy-related pattern through which supportive family processes may become connected to children’s learning dispositions.

In addition to the central role of parent-reported children’s self-efficacy, both the quantitative and qualitative findings indicate that children’s learning dispositions are associated with a multi-tiered motivational context, encompassing parenting self-efficacy, SES, and parenting arrangements. Within this context, the development of children’s learning dispositions appears to be more strongly associated with factors such as parenting self-efficacy and the quality of family interactional dynamics than directly by family structural conditions—a pattern consistent with Hypothesis 2, which posited a positive association between parenting self-efficacy and learning dispositions. However, the positive association between SES and learning dispositions, also proposed in Hypothesis 2, was significantly attenuated after controlling for parental involvement and parent-reported children’s self-efficacy; moreover, the node representing SES occupied a peripheral position in the network. Network analysis further revealed that SES was not directly connected with learning dispositions in the network but was connected primarily through, primarily through two pathways—parental involvement and parent-reported children’s self-efficacy. Second, the expectation that grandparental co-parenting would be negatively associated with learning dispositions was partially supported. One interpretation is that when grandparents become involved in the educational process and hold conflicting child-rearing beliefs, this may reduce consistency in rule enforcement, thereby undermining children’s self-regulatory capacities (Pittman & Boswell, 2007). Interview data further highlighted other informal support channels in this domain: emotionally supportive family environments were described as conducive to the accumulation of parent-reported children’s self-efficacy; some parents relied on friends or other parents for practical advice, suggesting that peer support can partially compensate for gaps in the family support system; and many parents lacking educational resources reported turning to short-video platforms for low-cost, easily digestible parenting information. In contrast, grandparental involvement in child-rearing was often perceived as a double-edged sword: while alleviating caregiving burdens, it may also weaken the consistency of educational approaches due to conflicting beliefs, thereby intensifying disagreements. In fact, previous research has shown that such co-parenting conflicts can further undermine parental competence and increase child problem behaviors (Pan et al., 2024). In some areas where filial norms remain deeply entrenched, some parents—particularly mothers—described feeling constrained when questioning grandparents’ child-rearing decisions. This situation sometimes led them to withdraw from daily educational responsibilities rather than engage more actively (Goh & Kuczynski, 2010).

In the domain of parental involvement, children’s learning dispositions were positively associated with parents’ educational engagement, consistent with Hypothesis 3. Quantitative results indicate a positive association between parental involvement and learning dispositions, with participation at home demonstrating the strongest correlation. Network analysis further reveals that, among the three dimensions of parental involvement, participation at home serves as a critical bridge connecting family contextual factors to children’s learning dispositions. One possible explanation is that participation at home, as the primary setting for daily parent–child interactions, encompasses diverse forms such as shared reading, exploratory activities in daily life, and practical hands-on interactions, thereby providing children with ample opportunities for experiential learning and emotional support (Fantuzzo et al., 2004). Interview data elucidate the logic and mechanisms through which participation at home links contextual factors to learning dispositions. Participation at home constitutes the primary context in which children accumulate mastery experiences, and such accumulation depends on the quality of involvement rather than the sheer amount of time spent—when work-related stress limits parents to superficial forms of accompaniment, children’s opportunities to receive efficacy-relevant feedback are correspondingly reduced. This observation aligns with prior research emphasizing the importance of the quality of parental involvement (Pomerantz et al., 2007; Fan & Williams, 2010). High-quality participation at home is often characterized by positive feedback, patient guidance, and bidirectional interaction, was associated with children displaying stronger “I can do it” confidence and greater willingness to persist as they complete tasks and overcome challenges, and was related to the formation and development of their learning dispositions (Gunderson et al., 2013). Furthermore, although participation at kindergarten and home–kindergarten communication were not directly associated with learning dispositions, they serve complementary functions in information calibration and strategy translation: the former allows parents to directly observe their children’s performance in group settings, capturing strengths and weaknesses that may not be evident at home; the latter establishes a bridge for collaboration between families and schools, translating teachers’ professional educational recommendations into concrete strategies that can be implemented within the home environment. In summary, within the influence of parental involvement on children’s learning dispositions, participation at home plays a central role in connecting and facilitating the process, working in conjunction with home–kindergarten communication and participation at kindergarten to form a support system that promotes the development of children’s learning dispositions.

Within the domain of child characteristics, children’s observed psychological characteristics may be associated with the formation and development of learning dispositions in preschoolers. Consistent with Hypothesis 1, quantitative results indicate a significant positive association between parent-reported children’s self-efficacy and learning dispositions, with self-efficacy serving as a central hub linking family factors to learning dispositions. Concurrently, qualitative analysis further reveals an important factor not explicitly included in the hypotheses—namely, emotional competence—which interacts with and mutually supports parent-reported children’s self-efficacy, functioning as an observed child-level process through which family resources may become linked to learning dispositions (Zuffianò et al., 2013). This finding resonates with existing empirical research showing that family processes, such as co-parenting quality, influence children’s developmental outcomes through their emotion regulation abilities (Pan et al., 2025; Wang et al., 2026). Interview data suggest that emotional competence appears to be an important psychological correlate of parent-reported children’s self-efficacy. Specifically, children with greater emotional stability tend to maintain focus and sustain effort when facing difficulties, thereby accumulating successful experiences more frequently. Parents described each successful experience as being followed by greater observable confidence and willingness to try, forming a cycle of “displayed confidence → attempt → success → stronger displayed confidence.” This observation aligns with previous research confirming the close association between emotional competence and self-efficacy, which together shape children’s learning dispositions (Bandura et al., 2003; Valiente et al., 2020). Moreover, parents adjust their involvement strategies in daily rearing practices based on children’s emotional states and behavioral performances: when children exhibit emotional distress or show signs of withdrawal in the face of challenges, parents increase guidance and emotional coaching; when children are emotionally stable and demonstrate initiative and creativity in learning-related activities, parents adopt a more tolerant and supportive role, allowing autonomous space for children’s self-development. In this sense, children’s emotional competence was associated with more robust parent-observed efficacy-related behaviors and, to some extent, was also described as being associated with parenting practices, thereby providing sustained internal motivation for the development of children’s learning dispositions.

Taken together, the three analytic strands support a proximal-process interpretation. The hierarchical regression showed that parent-reported children’s self-efficacy had the strongest standardized association with learning dispositions in the final model; the network analysis positioned it as a bridge connecting family- and child-level communities; and the qualitative interviews illustrated how daily interactions, mastery experiences, encouragement, and feedback were linked to children’s observable efficacy-related confidence. This convergence suggests that family resources and parental involvement may be most relevant to learning dispositions when they are translated into concrete learning experiences and supportive responses that children can enact behaviorally. At the same time, because the efficacy variable was reported by parents, these findings should be interpreted as evidence of parents’ perceptions of children’s efficacy-related behaviors, rather than as direct evidence of children’s internal beliefs.

### Limitations and future directions

Although this study contributes to understanding the correlates of young children’s learning quality, several limitations should be noted when interpreting the results, and these limitations also point to directions for future research.

First, although the quantitative sample of this study included four kindergartens in northern and southern China (*N* = 481) and the qualitative interviews involved 23 parents, the sample representativeness remains limited. The quantitative sample had a high proportion of mothers, which may limit the understanding of father involvement; although the qualitative sample considered diversity in region, educational level, and family structure, it could not cover all family types. Future research can verify the findings of this study in a larger and more diverse sample and recruit a more balanced sample of parents to examine whether the observed associations are applicable to fathers and different family care arrangements.

Second, all measurements were based on parental reports, which may be associated with common method bias and social desirability bias. For example, parents who self-report high involvement may systematically overestimate their children’s learning quality. Future research should incorporate multiple information sources and observational indicators, such as teacher evaluations, behavioral observations, and age-appropriate measurement tasks for young children, to enhance the reliability of evidence through data triangulation.

Third, the cross-sectional design limits the possibility of causal inference. Although this study used advanced statistical techniques such as hierarchical regression and network analysis to explore complex relationships, these methods cannot replace longitudinal evidence in establishing temporal order and excluding alternative explanations. Future research can adopt longitudinal designs or intervention studies to track how parent-reported children’s self-efficacy, parental involvement, and learning quality change together over time during early childhood.

Fourth, the bidirectional moderating relationships and boundary conditions revealed by qualitative analysis point to new directions for future research. On the one hand, cross-lagged designs can be used to track the long-term longitudinal associations between learning quality and parental involvement, testing reverse paths such as L4→L2 and L4→L1 that were not involved in the quantitative model. On the other hand, experience sampling methods can be used to examine the dynamic interaction between children’s emotional competence and parent-reported children’s self-efficacy in daily interactions, deepening the understanding of the mechanism that emotional competence serves as a supporting condition for self-efficacy. In addition, divergent cases suggest that attention should be paid to the moderating role of conditional variables (such as parental anxiety level, children’s temperamental characteristics, and early childcare experiences) on the model paths, to more accurately define the scope of application of the theoretical model.

Finally, although the qualitative analysis of this study deeply revealed the process through which parental involvement is associated with learning quality in Chinese families, caution is needed when generalizing the research results to other cultural contexts. There may be significant differences in family structure, educational concepts, and social support systems across different cultures. Future research can adopt cross-cultural comparative designs to explore the applicability and boundary conditions of the theoretical model of this study in different cultural contexts.

### Conclusions and implications

This study offers meaningful theoretical as well as applied implications for research on early childhood development and the pursuit of educational equity.

First, adopting a systemic perspective to examine the relationship between SES and young children’s learning dispositions, this study found that the two are not directly associated. Rather, they operate through a structured system with parent-reported children’s self-efficacy serving as the core integrative hub. In the network model, self-efficacy exhibited the highest bridge centrality, indicating that it connects previously disparate domains (family factors, parental involvement, and learning dispositions) and enables influences to propagate throughout the system. Initiative within learning dispositions and participation at home within parental involvement also played important connecting roles; however, parent-reported children’s self-efficacy occupied the most strategic position. The qualitative analysis further revealed the underlying mechanisms of this hub position: factors such as family climate, parental involvement, and teacher feedback, through daily immersion and concrete interactions, were described as helping children display “I can do it” confidence and efficacy-related behaviors, which were further linked to the development of learning dispositions qualities such as curiosity and interest, initiative, and persistence.

Second, the findings carry significant equity-oriented implications for China and other developing or rapidly transforming societies facing widening socioeconomic disparities. Since SES is associated with learning dispositions primarily indirectly through family processes and parent-reported children’s self-efficacy, rather than through direct effects, policies focusing solely on institutional inputs are insufficient to bridge the gap. Effective strategies should be family-centered and efficacy-focused. The qualitative analysis revealed that when parents possess higher parenting self-efficacy, the family atmosphere is relaxed and loving, and grandparents provide collaborative support, parents described children as more likely to accumulate successful experiences and display “I can do it” confidence within a stable and secure environment.

In conclusion, young children’s learning dispositions are associated with the interplay between children’s observable efficacy-related motivation and malleable family processes, which are, in turn, embedded within broader socioeconomic structures. Identifying parent-reported children’s self-efficacy as a proximal child-level indicator and participation at home as the primary carrier helps redirect research and policy toward developmentally sensitive, family-embedded, and contextually responsive interventions, enabling all children—regardless of their backgrounds—to obtain a solid and equitable starting point.

## Supplementary Information

Supplementary Material 1.

## Data Availability

The datasets generated and/or analysed during the current study are not publicly available due to participant privacy and ethical restrictions but are available from the corresponding author upon reasonable request.
