# Supplementary material for: Parent-reported children’s self-efficacy in linking family resources to preschoolers’ learning dispositions: a mixed-methods study
Source: Psicol Reflex Crit. 2026 May 19;39:26. doi: 10.1186/s41155-026-00397-y (PMC13365098; doi:10.1186/s41155-026-00397-y)
Supplement: Supplementary file 1 — Supplementary Material 1. [file 41155_2026_397_MOESM1_ESM.docx]

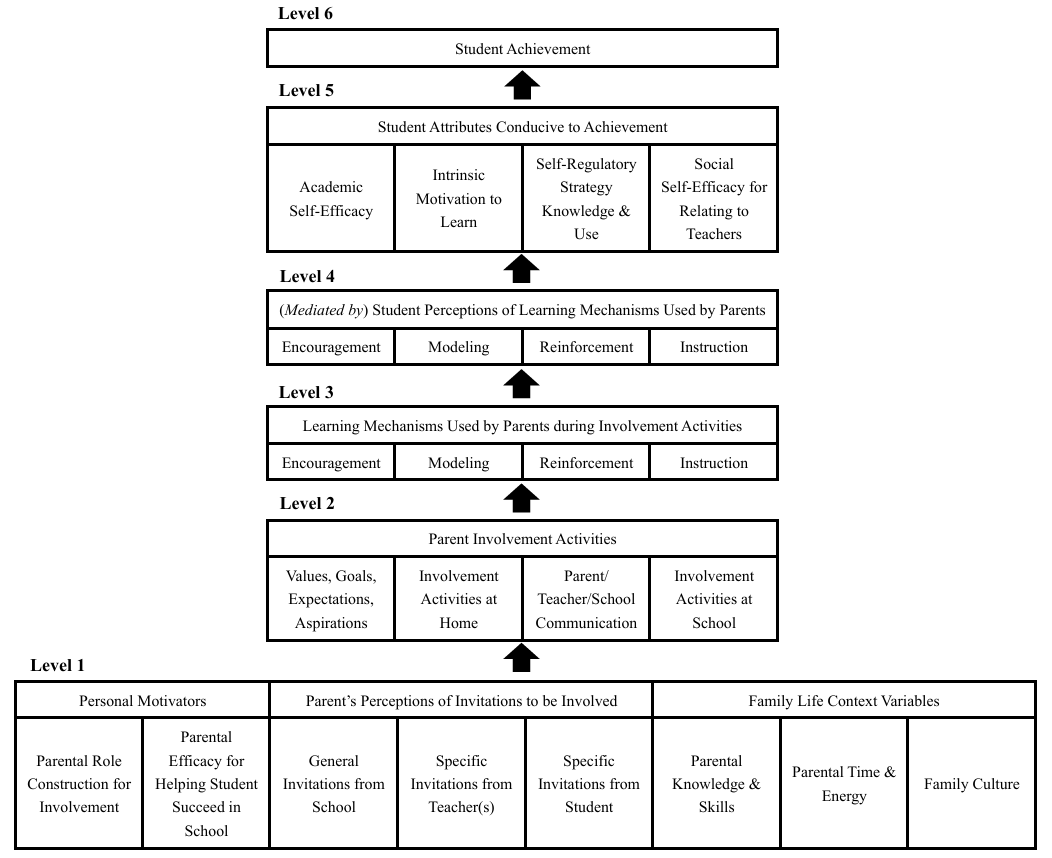


**Figure S1.** Model of the Parental Involvement Process.


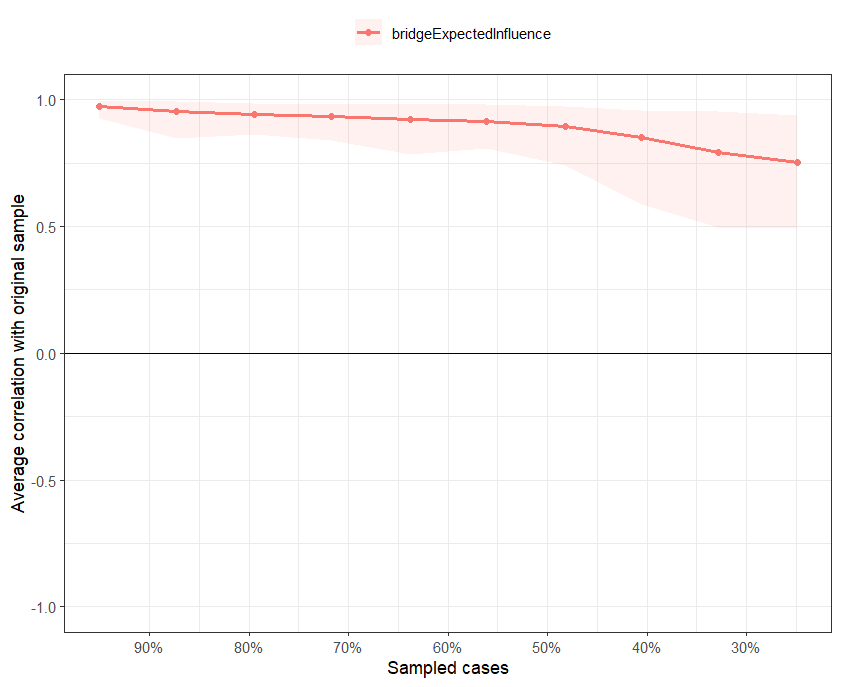


**Figure S2.** Bridge expected influence stability plot.


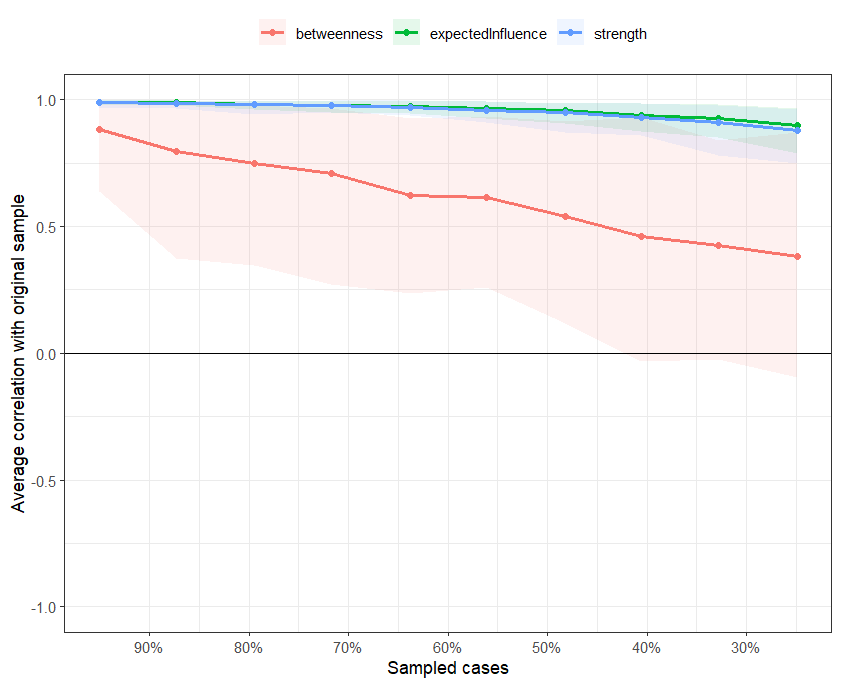


**Figure S3.** Stability of node centrality indices.


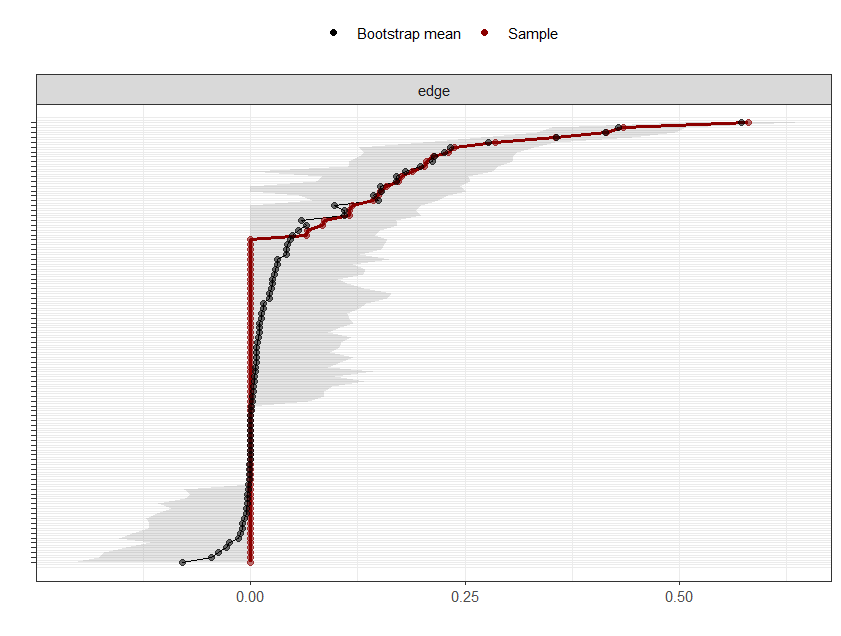


**Figure S4.** Edge-weight stability for the full-sample network is shown here. Each horizontal line corresponds to one edge; the point estimate is indicated by the red line, whereas the grey band denotes the 95% confidence interval.

**Table S1** List of interview subjects (*N*=23).

| ID | Gender | Educational Level | Parents’ Age | Occupation | Income | Childs’ Age | Family Structure | Duration (min) |
| --- | --- | --- | --- | --- | --- | --- | --- | --- |
| T1 | Female | Bachelor’s degree | 34 | Primary school teacher | 5,001–10,000 | 5 | Stem family | 42 |
| T2 | Female | Associate degree | 31 | Unemployed | 2,001–3,000 | 4 | Stem family | 38 |
| T3 | Male | High school | 36 | Self-employed | 4,001–5,000 | 6 | Nuclear family | 41 |
| T4 | Female | Master’s degree | 33 | Secondary school teacher | 10,001–20,000 | 4 | Stem family | 45 |
| T5 | Female | High school | 29 | Temporary worker | 2,001–3,000 | 3 | Stem family | 37 |
| T6 | Female | Bachelor’s degree | 35 | Kindergarten teacher | 5,001–10,000 | 5 | Stem family | 44 |
| T7 | Female | Associate degree | 38 | Self-employed | 4,001–5,000 | 6 | Stem family | 40 |
| T8 | Male | Middle school | 40 | Agricultural laborer | 3,001–4,000 | 5 | Stem family | 36 |
| T9 | Female | Bachelor’s degree | 32 | Unemployed | 3,001–4,000 | 4 | Stem family | 43 |
| T10 | Female | Associate degree | 30 | Kindergarten teacher | 4,001–5,000 | 4 | Stem family | 39 |
| T11 | Female | Master’s degree | 37 | High school teacher | 10,001–20,000 | 6 | Nuclear family | 48 |
| T12 | Female | Bachelor’s degree | 33 | Self-employed | 5,001–10,000 | 4 | Stem family | 41 |
| T13 | Female | High school | 39 | Unemployed | 2,001–3,000 | 5 | Stem family | 38 |
| T14 | Male | Associate degree | 35 | Self-employed | 4,001–5,000 | 4 | Stem family | 40 |
| T15 | Female | Bachelor’s degree | 31 | Primary school teacher | 5,001–10,000 | 3 | Stem family | 43 |
| T16 | Female | High school | 41 | Temporary worker | 2,001–3,000 | 6 | Stem family | 39 |
| T17 | Female | Associate degree | 34 | Self-employed | 4,001–5,000 | 5 | Stem family | 42 |
| T18 | Male | Bachelor’s degree | 36 | Training instructor | 5,001–10,000 | 4 | Stem family | 44 |
| T19 | Female | Vocational school | 28 | Unemployed | 2,001–3,000 | 3 | Stem family | 35 |
| T20 | Female | Bachelor’s degree | 35 | Kindergarten teacher | 5,001–10,000 | 5 | Nuclear family | 41 |
| T21 | Female | Associate degree | 33 | Self-employed | 4,001–5,000 | 4 | Stem family | 40 |
| T22 | Male | High school | 42 | Temporary worker | 3,001–4,000 | 6 | Stem family | 38 |
| T23 | Female | Bachelor’s degree | 32 | Primary school teacher | 5,001–10,000 | 3 | Stem family | 42 |

| **Variables** | PE1 | PE2 | PE3 | SES | PA | PI1 | PI2 | PI3 | SE | LD1 | LD2 | LD3 | LD4 | LD5 |
| --- | --- | --- | --- | --- | --- | --- | --- | --- | --- | --- | --- | --- | --- | --- |
| PE1 | - |  |  |  |  |  |  |  |  |  |  |  |  |  |
| PE2 | 0.580 | - |  |  |  |  |  |  |  |  |  |  |  |  |
| PE3 | 0.000 | 0.285 | - |  |  |  |  |  |  |  |  |  |  |  |
| SES | 0.000 | 0.000 | 0.000 | - |  |  |  |  |  |  |  |  |  |  |
| PA | 0.000 | 0.000 | 0.000 | 0.000 | - |  |  |  |  |  |  |  |  |  |
| PI1 | 0.000 | 0.000 | 0.000 | 0.000 | 0.000 | - |  |  |  |  |  |  |  |  |
| PI2 | 0.000 | 0.000 | 0.000 | 0.000 | 0.000 | 0.415 | - |  |  |  |  |  |  |  |
| PI3 | 0.086 | 0.000 | 0.000 | 0.000 | 0.000 | 0.158 | 0.238 | - |  |  |  |  |  |  |
| SE | 0.000 | 0.000 | 0.189 | 0.000 | 0.000 | 0.000 | 0.000 | 0.000 | - |  |  |  |  |  |
| LD1 | 0.000 | 0.000 | 0.000 | 0.000 | 0.000 | 0.000 | 0.000 | 0.000 | 0.231 | - |  |  |  |  |
| LD2 | 0.000 | 0.000 | 0.143 | 0.000 | 0.000 | 0.000 | 0.065 | 0.066 | 0.202 | 0.205 | - |  |  |  |
| LD3 | 0.000 | 0.118 | 0.000 | 0.000 | 0.000 | 0.000 | 0.000 | 0.213 | 0.000 | 0.116 | 0.149 | - |  |  |
| LD4 | 0.000 | 0.000 | 0.000 | 0.000 | 0.000 | 0.000 | 0.000 | 0.083 | 0.000 | 0.151 | 0.355 | 0.172 | - |  |
| LD5 | 0.000 | 0.000 | 0.000 | 0.000 | 0.000 | 0.000 | 0.000 | 0.000 | 0.000 | 0.434 | 0.176 | 0.000 | 0.115 | - |

**Table S2** Edge-Weight Matrix for the Network Plot.

**Table S3** Open coding (part).

| **Original Statement (Examples)** | **Initial Concept** | **File Count** | **Reference Points** | **Category** |
| --- | --- | --- | --- | --- |
| T13: The grandparents do not experience the same level of anxiety as our generation, which also has a positive influence on the child’s way of handling things.  T13: My father-in-law told me that if the child wants to do something, we should let him persevere on his own, and that it is normal for a young child to cry and whine at the beginning. | Grandparental support (positive) | 3 | 5 | Grandparental support |
| T11: The grandparents are quite doting towards the child and tend to be overprotective in daily life, for instance, by wanting to feed him during meals.  T15: When we criticize him in front of his grandparents, he doesn’t seem as afraid, because he feels that his grandparents can come to his aid. | Grandparental support (negative) | 9 | 11 |  |
| T2: The grandparents are mainly responsible for picking up and dropping off the child and preparing meals. However, tasks such as playing with the child at home and providing guidance are mostly handled by us, with minimal involvement from the grandparents. | Grandparental support (neutral) | 7 | 7 |  |
| …… |  |  |  |  |
| T11: He is now able to arrange his own schedule. When he comes home in the evening, he plans which online classes to take, what homework to do, and what books to read. I just need to sit by his side.  T1: He started attending nursery school when he was about one year and ten months old. He had to learn to speak, communicate, and express things clearly at a very young age, which has also been quite helpful in fostering his independence. | Independence | 4 | 6 | Independence- Dependence |
| T12: I am essentially a “kangaroo mother”; he has never really been away from me.  T2: He is quite dependent on us. If I am the one taking him to school, he cries. | Dependence | 1 | 1 |  |
| T5: When faced with simple tasks, he would say, “This is easy, I can do it.”  T6: When he does well in his studies, he also praises himself, saying he is great. | Positive self-evaluation | 13 | 34 | self-evaluation |
| T1: He says he can’t read well himself. When he gets to a difficult part while reading, he develops a kind of obsession with it, then starts to cry and doubt himself.  T8: She would say dejectedly, “I’m so useless, I can’t do anything right.” At such times, it takes a lot of time to comfort and encourage her. | Negative self-evaluation | 2 | 2 |  |
| T6: She is interested in everything and willing to explore new things. For example, this semester she took the initiative to say she wanted to learn dance.  T14: During this period, probably after reading some books, he has developed a strong curiosity in daily life. | Curiosity and interest | 2 | 2 | Learning dispositions |
| T2: He is willing to take the initiative to try things, and sometimes he even proactively grabs me to engage in spoken dialogue with him. | Initiative | 3 | 5 | Learning dispositions |
| …… |  |  |  |  |

Note: In order to save space, only part of the categorisation process is shown in this table.

**Table S4** Spindle coding.

| Category | File Count | Reference Points | Main Category | File Count | Reference Points |
| --- | --- | --- | --- | --- | --- |
| B1 Educational philosophy | 13 | 25 | Level 1: Motivational and Situational Foundations | 16 | 85 |
| B2 Parenting self-efficacy | 5 | 6 |  |  |  |
| B3 Grandparental support | 16 | 23 |  |  |  |
| B4 Socioeconomic status (SES) | 11 | 41 |  |  |  |
| B5 Family atmosphere | 8 | 9 |  |  |  |
| B6 Community support | 1 | 1 |  |  |  |
| B7 Online resources | 1 | 1 |  |  |  |
| B8 Peer support | 1 | 1 |  |  |  |
| B9 School support | 4 | 4 |  |  |  |
| B10 Home-kindergarten communication | 10 | 11 | Level 2: Parental Involvement | 14 | 63 |
| B11 Kindergarten-based involvement | 1 | 1 |  |  |  |
| B12 Home-based involvement | 14 | 51 |  |  |  |
| B13 Independence | 4 | 6 | Level 3: Child Characteristics | 14 | 71 |
| B14 Dependence | 1 | 1 |  |  |  |
| B15 Difficulty coping patterns | 5 | 7 |  |  |  |
| B16 Task initiation modes | 3 | 7 |  |  |  |
| B17 Emotion understanding | 2 | 2 |  |  |  |
| B18 Emotion regulation | 5 | 6 |  |  |  |
| B19 Emotion expression | 2 | 5 |  |  |  |
| B20 Emotion awareness | 5 | 6 |  |  |  |
| B21 Curiosity and interest | 2 | 2 | Level 4: Learning Dispositions | 14 | 21 |
| B22 Initiative | 3 | 5 |  |  |  |
| B23 Persistence | 5 | 5 |  |  |  |
| B24 Concentration | 6 | 7 |  |  |  |
| B25 Reflective ability | 2 | 2 |  |  |  |

**Table S5.** Joint Display of Quantitative and Qualitative Findings

| **Domain** | **Quantitative Findings** | **Qualitative Findings** | **Meta-Inferences** |
| --- | --- | --- | --- |
| Level 1: Motivational and Situational Foundations | Parenting self-efficacy was the strongest predictor in Model 1 (*β* = 0.688, *p* < 0.001), remaining significant after adding other variables (Model 3 *β* = 0.298, *p* < 0.001). SES was significant in Model 1 (*β* = 0.126, *p* < 0.001) but weakened after parental involvement was introduced (Model 3 *β* = 0.068, *p* < 0.01). Network analysis showed SES and parenting arrangements were peripheral (strength = 0), while parenting self-efficacy connected directly to children’s self-efficacy. | Parents with high self-efficacy provided supportive companionship, helping children accumulate success experiences; those with low self-efficacy felt helpless, and their children gave up more easily. Grandparental support had dual effects: when aligned with parental authority, it freed time for quality involvement; when conflicting, it undermined parental authority and children’s rule awareness. Family atmosphere provided emotional security; social support systems (community, teachers, peers) offered informational and emotional resources. | Expansion. Quantitative effects of SES diminished after parental involvement was added; qualitative data explain this: SES operates through time and energy resources affecting involvement quality, not directly on learning dispositions. The dual effect of grandparental support explains its peripheral but significant negative association—the key is authority alignment, not grandparent presence per se. |
| Level 2: Parental Involvement | Parental involvement significantly predicted learning dispositions (Model 2 *β* = 0.356, *p* < 0.001; Model 3 *β* = 0.286, *p* < 0.001). Network analysis identified home-based involvement (PI3) as a key bridge node (bridge strength = 0.45), connecting contextual factors, self-efficacy, and learning dispositions. Home-kindergarten communication and kindergarten-based involvement showed weaker connections. | Home-based involvement was the most central form, supporting children’s self-efficacy through daily interaction. Home-kindergarten communication was primarily event-driven; parents hesitated to seek everyday advice for fear of “bothering” teachers. Kindergarten-based involvement was constrained by work schedules and perceptions of kindergarten functions (educational vs. custodial). | Confirmation. Quantitative centrality of home-based involvement is explained by qualitative data: it serves as the primary setting for efficacy cultivation. The peripheral position of other involvement forms reflects rural families' time poverty and limited teacher communication. |
| Level 3: Child Characteristics | Children’s self-efficacy was the strongest predictor in Model 3 (*β* = 0.385, *p* < 0.001). Network analysis showed self-efficacy (SE) had the highest bridge strength (0.62) and bridge expected influence (0.62), serving as the core hub connecting all domains. Emotional competence correlated with self-efficacy (*r* = 0.492, *p* < 0.001) and connected to learning dispositions in the network. | Self-efficacy was the core mechanism transforming family resources and parental involvement into learning dispositions. Parental involvement helped children accumulate success experiences, building “I can do it” beliefs; positive teacher feedback, when transmitted by parents, reinforced domain-specific efficacy. Emotional competence supported self-efficacy: emotionally stable children maintained efficacy beliefs when facing difficulties; family atmosphere and parental emotional stability shaped children's emotional competence. | Confirmation and hub positioning. Quantitative data position self-efficacy as the system's core bridge node; qualitative data reveal its mechanism: transforming family support and parental involvement into children's intrinsic motivation. Emotional competence explains why some children with similar family backgrounds differ in learning dispositions—emotion regulation affects efficacy maintenance and recovery. |
| **Level 4: Learning Dispositions** | The final model explained 69.5% of variance in learning dispositions (*R*² = 0.695). Network analysis showed strong internal associations among dimensions; initiative (LD2) had the highest strength (1.36) and expected influence (1.36), making it the most central node. Other dimensions connected to parental involvement through self-efficacy. | Parents described learning dispositions as multidimensional: curiosity, initiative, persistence, concentration, and reflective ability. These dimensions were closely tied to children's self-efficacy—children with stronger efficacy were more willing to try new things, persist through difficulties, and learn from experience. | Integration. Quantitative data confirm the multidimensional structure of learning dispositions and identify initiative as the core dimension; qualitative data validate the connection between these dimensions and self-efficacy. Together, they support the theoretical pathway: Level 1 → Level 2 → Level 3 → Level 4. |
